# Characterization of *Arabidopsis* Transcriptional Responses to Different Aphid Species Reveals Genes that Contribute to Host Susceptibility and Non-host Resistance

**DOI:** 10.1371/journal.ppat.1004918

**Published:** 2015-05-20

**Authors:** Maëlle Jaouannet, Jenny A. Morris, Peter E. Hedley, Jorunn I. B. Bos

**Affiliations:** 1 Cell and Molecular Sciences, The James Hutton Institute, Dundee, United Kingdom; 2 Dundee Effector Consortium, Dundee, United Kingdom; 3 Division of Plant Sciences, College of Life Sciences, University of Dundee, Dundee, United Kingdom; Boyce Thompson Institute, UNITED STATES

## Abstract

Aphids are economically important pests that display exceptional variation in host range. The determinants of diverse aphid host ranges are not well understood, but it is likely that molecular interactions are involved. With significant progress being made towards understanding host responses upon aphid attack, the mechanisms underlying non-host resistance remain to be elucidated. Here, we investigated and compared *Arabidopsis thaliana* host and non-host responses to aphids at the transcriptional level using three different aphid species, *Myzus persicae*, *Myzus cerasi* and *Rhopalosiphum pisum*. Gene expression analyses revealed a high level of overlap in the overall gene expression changes during the host and non-host interactions with regards to the sets of genes differentially expressed and the direction of expression changes. Despite this overlap in transcriptional responses across interactions, there was a stronger repression of genes involved in metabolism and oxidative responses specifically during the host interaction with *M*. *persicae*. In addition, we identified a set of genes with opposite gene expression patterns during the host versus non-host interactions. Aphid performance assays on *Arabidopsis* mutants that were selected based on our transcriptome analyses identified novel genes contributing to host susceptibility, host defences during interactions with *M*. *persicae* as well to non-host resistance against *R*. *padi*. Understanding how plants respond to aphid species that differ in their ability to infest plant species, and identifying the genes and signaling pathways involved, is essential for the development of novel and durable aphid control in crop plants.

## Author Summary

Aphids are phloem-feeding insects that cause feeding damage and transmit plant viruses to many crops. While most aphid species are restricted to one or few host plants, some aphids can infest a wide range of plant species. These insects spend a considerable time on non-hosts, where they probe the leaf tissue and secrete saliva, but for unknown reasons are unable to ingest phloem sap. This suggests that aphids interact with non-host plants at the molecular level, but potentially do not suppress plant defences and/or promote the release of nutrients. We compared gene expression of plants during host and non-host interactions with aphids to identify genes involved in immunity. We found significant overlap in the plant responses to aphids regardless of the type of interaction. Despite this, we identified a set of genes specifically affected during host or non-host interactions with specific aphid species. In addition, we showed that several of these genes contribute to host and/or non-host immunity. These findings are important, as they advance our understanding of the plant cellular processes involved in host and non-host responses against insect pests. Understanding mechanisms of host and non-host resistance to plant parasites is essential for development of novel control strategies.

## Introduction

Aphids are hemipteran insects that display exceptional variation in host range. While some aphid species, such as *Myzus persicae* (green peach aphid), are able to infest plants in over 40 families, including many important crops, closely related *Myzus cerasi* (black cherry aphid) is only able to infest a limited number of hosts within one or two plant families [1]. Other species, for example *Rhopalosiphum padi* (bird-cherry oat aphid), are limited to cereal crops. The underlying mechanism of aphid host range is not well understood, but is likely determined by complex molecular interactions in both host and aphid species. Upon landing on a leaf surface, insects perceive several types of plant structures and volatiles that can indicate host suitability [2]. On host plants, aphids are able to establish a phloem-feeding site upon probing, using their specialized mouthparts called stylets. However, it has been reported that aphids exhibit probing behavior regardless of the plant species they land on, and thus regardless of host suitability [3,4,5,6]. Interestingly, it has been suggested that aphids have increased probing rates in non-host interactions, which explains the higher virus transmission rates by aphids reported on non-host plant species [2]. These observations imply that there is an opportunity for molecular interactions to take place during both aphid-host and non-host interactions. Perhaps as a results of these molecular interactions, aphids are either unable to reach the phloem or unable to successfully feed from the phloem of non-host plants.

To date most research with regards to plant defence signaling has been focused on compatible interactions, and in particular on the *Arabidopsis thaliana*–*M*. *persicae* interaction. As reviewed in detail by Louis and Shah [7], SA (salicylic acid)-, JA (jasmonic acid)-, ET (ethylene)- and ABA (abscisic acid)-signaling pathways are all involved in host defence responses against aphids, but their exact role is still not clear and may vary among plant species. Secondary metabolites are also known to be important in host defences against aphids. For example, PAD4 (phytoalexin-deficient 4), a lipase-like protein, and PAD3, a cytochrome P450 that is involved in formation of camalexin, are both important in *Arabidopsis* defences against *M*. *persicae* [8], [9], [10]. Also, glucosinolates, which increase upon aphid feeding, reduce *Arabidopsis* susceptibility to aphids [11].

More recently, evidence for the involvement of PAMP (Pathogen Associated Molecular Pattern)-triggered immunity (PTI) in plant-aphid interactions has emerged. Work by Prince et al. [12] showed that BAK1 (Brassinosteroid insensitive 1-associated receptor kinase 1), which functions as a co-receptor for PRRs (pattern recognition receptors) to trigger PTI, may be involved in non-host resistance to aphids. More specifically, survival rates of *Acyrthosiphon pisum* (pea aphid) were increased on *Arabidopsis bak1-5* mutants compared to wild type plants three to four days upon aphid challenge suggesting that BAK1 contributes to non-host resistance. Although it is possible that unidentified PRRs recognize conserved aphid molecules to trigger PTI, molecules from aphid-associated organisms such as bacteria, viruses or fungi could also be recognized. Bacterial GroEL is present in aphid saliva, among several other bacterial proteins, and activates PTI-like responses that reduce aphid virulence [13], [14]. Another layer of defences involved in aphid recognition involves NB-LRR (nucleotide-binding site leucine-rich repeat) proteins. In several plant-aphid systems, resistance (R) proteins have been identified that confer resistance to specific aphid biotypes and have a typical CC-NB-LRR structure, similar to R proteins conferring resistance to plant pathogens [15].

Another plant response activated during the interaction with herbivorous insects is the production of Reactive Oxygen Species (ROS). *Diuraphis noxia* (Russian Wheat Aphid) triggers production of ROS in resistant wheat lines, while a slight increase was also observed in susceptible lines [16], which may reflect the activation of a hypersensitive response (HR). However, several studies in dicots have indicated ROS-signaling also is activated in compatible interactions. *Arabidopsis* gene expression analyses upon infestation with *Brevicoryne brassicae* (cabbage aphid) showed differential expression changes of genes involved in the oxidative stress response and the generation of ROS as early as 6 hours post aphid challenge and highest expression of these genes 24 hours post insect challenge [17]. Kerchev et al. [18] provided evidence for activation of oxidative responses in the potato-*M*. *persicae* interaction 48 hours after challenging plants with aphids. Also in pea, an oxidative response, including the production of ROS, was observed upon host interaction with *A*. *pisum* [19]. By using the dye DCFH-DA (dichlorodihydro-fluorescein diacetate), the ROS burst associated with aphid attack was observed and showed a peak in ROS production at 24 hours post aphid challenge.

The production of ROS upon plant parasite attack involves NADPH-oxidases. In *Arabidopsis* at least two NADPH-oxidase isoforms, AtRbohD and AtRbohF, are involved in the production of ROS upon interaction with an avirulent *Pseudomonas syringae pv*. *tomato* strain and the oomycete pathogen *Hyaloperonospora parasitica* [20]. While AtRbohD plays a more pronounced role in ROS production than AtRbohF, the latter shows more involvement in the control of cell death triggered by plant pathogens. Interestingly, AtRbohD contributes to plant defences against aphids as reflected by increased susceptibility of the *atrbohD-3* knockout mutant to *M*. *persicae* [21]. Moreover, this mutant also showed reduced ROS levels upon treatment with aphid-derived extract containing elicitor(s) [12]. Whether AtRbohF also contributes to plant defences against aphids, and whether AtRbohD and AtRbohF are involved in non-host responses to aphids remains to be investigated.

Nonhost resistance to plant pathogens involves recognition events and activation of plant immunity, which can be suppressed and/or evaded by effector repertoires in compatible interactions [22], [23]. Although important progress has been made in understanding how plants respond to aphids in compatible interactions, there is a need to investigate and compare how plants respond to aphids during non-host interactions. Here, we aimed to characterize *Arabidopsis* responses during host and non-host interactions with three different aphid species, *M*. *persicae*, *M*. *cerasi* and *R*. *padi*. *Arabidopsis* is not considered a host for *M*. *cerasi* and *R*. *padi* based on available literature, but is a host for the broad host range aphid *M*. *persicae*. To gain insight into overall plant responses to these aphids, we performed transcriptome analyses, which revealed high levels of similarity in *Arabidopsis* transcriptional changes as a consequence of the different aphid interactions, with the exception of a relatively small set of genes. We used the transcriptome data to select genes for further characterization with regards to their contribution to plant-aphid interactions and identified several genes involved in host susceptibility to *M*. *persicae* and *M*. *cerasi* and non-host resistance to *R*. *padi*.

## Results

### Different levels of *Arabidopsis* colonization by *M*. *persicae*, *M*. *cerasi* and *R*. *padi*

**Fig 1:**
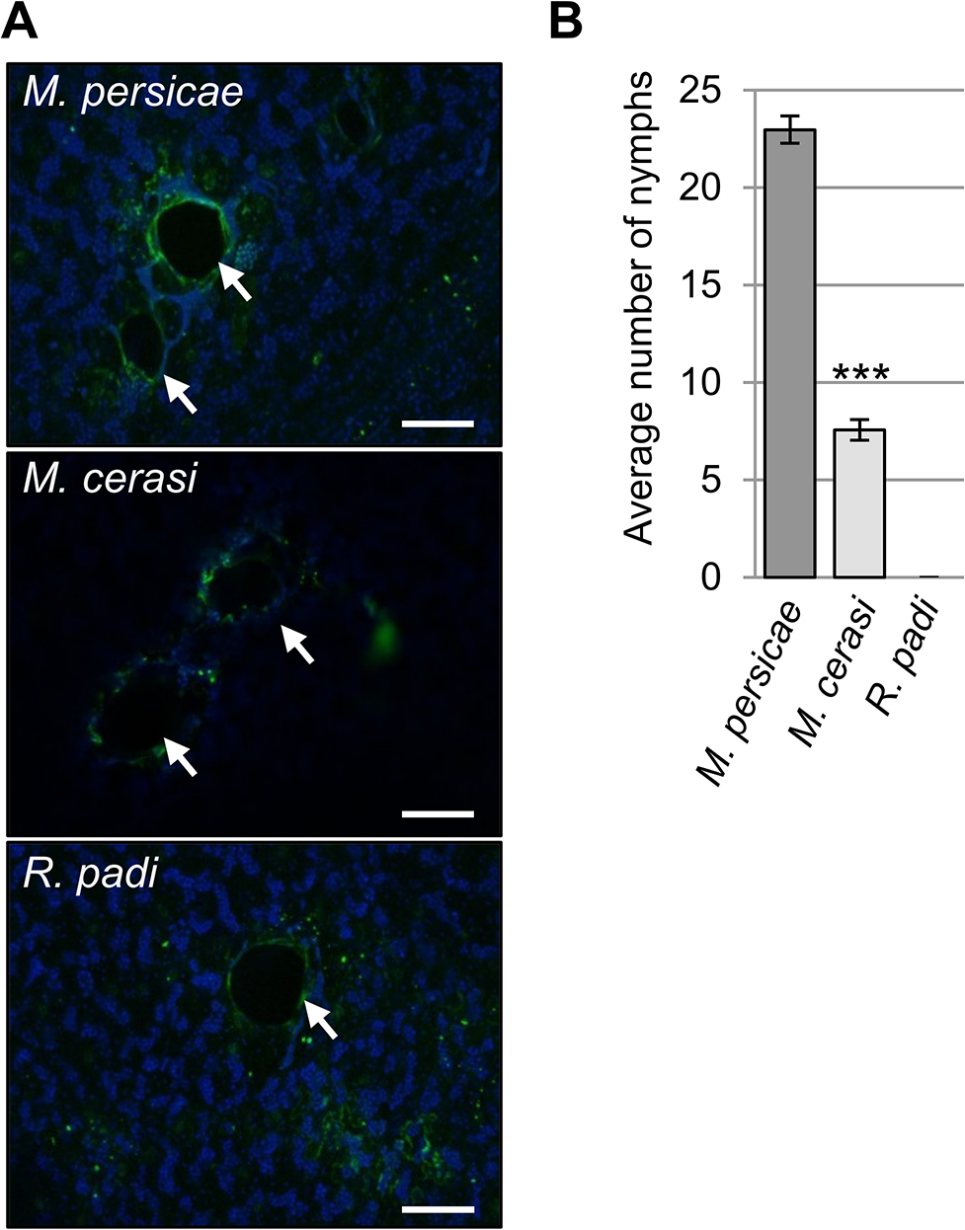
Aphid probing during host and non-host interactions. (A) Autofluorescence around aphid probe sites, indicated by white arrows, was visualized using laser confocal microscopy. Scale bars 100μm. (B) Aphid colonization of *Arabidopsis* by *Myzus persicae*, *M*. *cerasi* and *Rhopalosiphum padi*. Graph shows the mean number of nymphs produced after two weeks on wild type Col-0 plants. Error bars indicate standard error. A Student’s *t*-test was used for statistical analysis of *M*. *persicae* versus *M*. *cerasi* progeny (*** indicates p-value < 0.001). Three independent biological replicates were carried out, with 10 plants per treatment per replicate.

Aphid probing generally takes place in non-host interactions and is responsible for the high transmission rates of viruses by aphids on non-host plant species (Harrington et al., 1986). To test whether *Myzus cerasi* and *Rhopalosiphum padi* probe the leaf surface during the interaction with *Arabidopsis* we assessed leaves challenged with these aphid species as well as *Myzus persicae* for the presence of autofluorescence, indicative of damaged epidermal cells. This showed that indeed probing takes place during the different interactions with aphids by the presence of puncture sites, surrounded by autofluorescence (Fig 1A). In addition, we performed acid fuchsin staining, which provides a pink staining of aphid stylet sheath proteins, to visualize aphid stylet pathways in leaf tissue. This confirmed stylet pathways were present in *Arabidopsis* leaves upon challenge with the three different aphid species (S1 Fig). Finally, we used trypan blue staining to visualize plant cell death. This showed that all aphid species were able to cause cell death, either due to damage or activation of plant defences, during the interaction (S1 Fig). Importantly, these observations indicate that transient but yet intimate associations take place in both host and non-host interactions that allow for molecular interactions to occur.

Unexpectedly, while performing the aphid probing assays we noticed that *M*. *cerasi* was able to reproduce on *Arabidopsis*. To further determine and compare the colonization rates of the different aphid species on *Arabidopsis* we allowed aphids to infest plants over a 14-day period, starting with 2 (age-synchronized) adults on day one. Fourteen days later the total aphid population per plant was counted, including all adults and nymphs. For *M*. *persicae*, the population consisted on average of 23 aphids per plant, and as expected, *R*. *padi* was unable to survive and reproduce (Fig 1B). Remarkably, the *M*. *cerasi* population consisted of around 8 aphids per plant, indicating that under our growth room conditions this species was able to colonize *Arabidopsis* to a relatively low level compared to *M*. *persicae* (Fig 1B). Similar infestation experiments of cress plants showed the *M*. *cera*si population on this host plant consisted of around 28 aphids on average (S1 Fig). Although *M*. *cerasi* has not been reported on *Arabidopsis*, our observation suggests that this aphid is able to utilize this plant species as a host under greenhouse conditions. Potential host ranges as determined under laboratory conditions have been reported to differ from actual host ranges in the field for several insect pests, which may reflect the impact of environmental factors on plant susceptibility and insect behavior and predation [24,25,26]. We will refer to the *Arabidopsis*-*M*. *cerasi* interaction as a “poor-host interaction” in this manuscript.

### *Arabidopsis* transcriptome analyses reveal an enhanced response to interaction with *M*. *persicae* versus interaction with *M*. *cerasi* and *R*. *padi*

**Fig 2:**
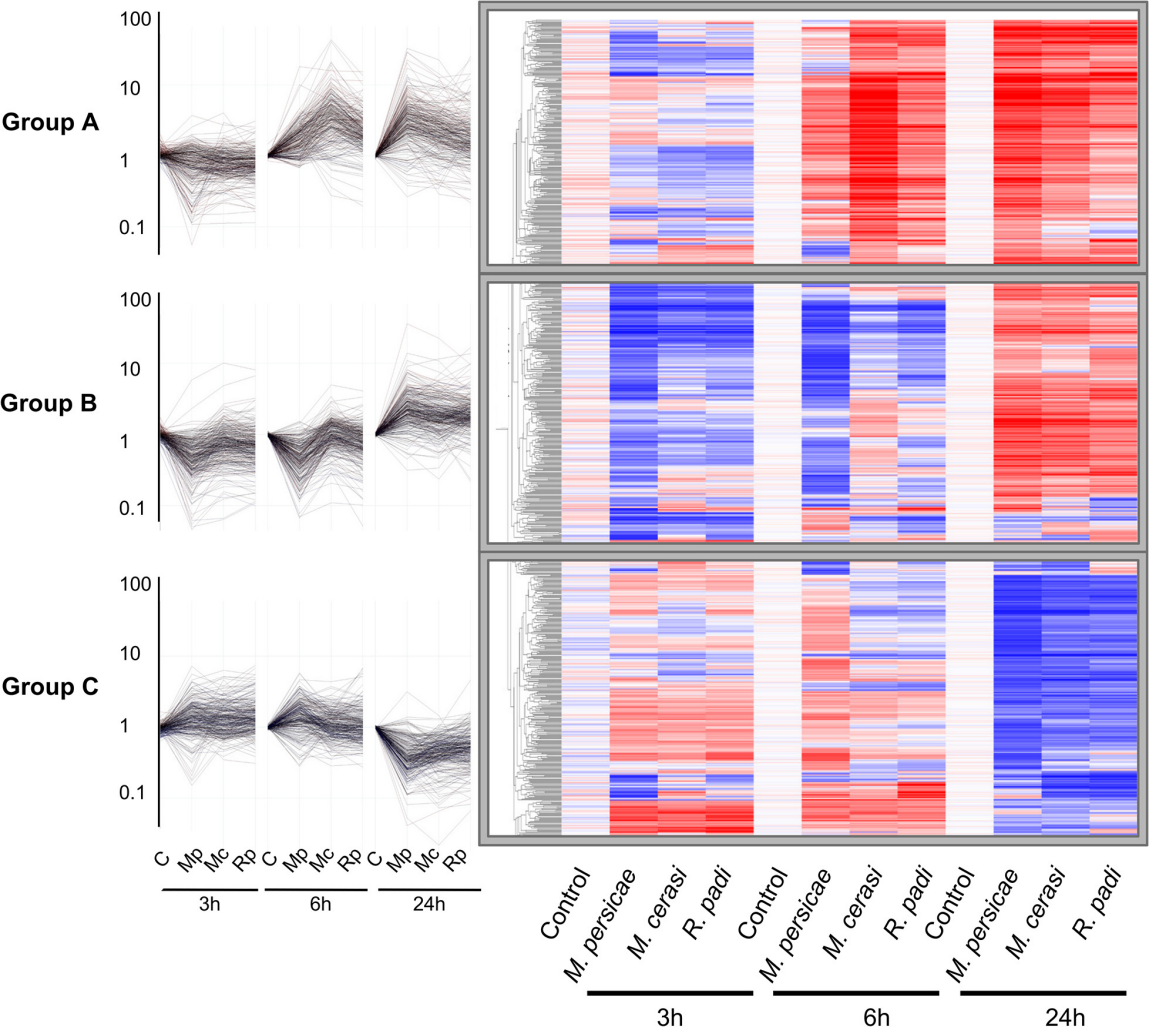
Clustering of 874 differentially expressed *Arabidopsis* genes during host and non-host interactions with aphids. Using one-way ANOVA in GeneSpring (Bonferroni correction, p-value ≤ 0.05), we identified 874 genes that display significant differential expression across different aphid treatments and timepoints. Hierarchical gene tree cluster analysis of these 874 genes using GeneSpring software identified three main clusters of genes (A, B and C) according to their expression profiles across all treatments and timepoints. Low gene expression levels are indicated by blue colour and high gene expression levels are indicated by red colour. Mc indicates *Myzus cerasi*, Mp indicates *M*. *persicae* and Rp indicates *Rhopalosiphum padi*.

To further investigate *Arabidopsis* host, poor-host, and non-host responses to *M*. *persicae*, *M*. *cerasi* and *R*. *padi*, respectively, we performed microarray analyses using Agilent *Arabidopsis* 4×44K arrays. Plants were challenged with the different aphid species and above ground plant tissues were harvested after 0, 3, 6 and 24 hours. We identified 874 genes that displayed significant differential expression in at least one of the aphid treatments compared to the no-aphid control. Based on the gene expression profiles of these genes, we identified three main gene clusters (Fig 2). Cluster A groups together 275 genes up-regulated at 6h and 24h, cluster B comprises 306 genes up-regulated at 24h and mostly down-regulated at 3 and 6 hours, and cluster C contains 293 genes that are mainly down-regulated at 24h (Fig 2, S1 Table). Interestingly, the overall transcriptome changes with regard to direction of changes are quite similar among different aphid treatments. However, at 3h and 6h, the downregulation of a number of genes in cluster A and B is more pronounced during the host interaction with *M*. *persicae* than during the poor-host or non-host interactions with *M*. *cerasi* or *R*. *padi*, respectively. We performed Gene Ontology (GO) analyses of the genes within the three different clusters to assess whether there was an association with specific predicted gene functions. For cluster A, the main predicted gene functions were in transcriptional processes, and for cluster B main functions were related to metabolism, including ROS metabolism (S2 Table). Although there was no obvious main functional category for cluster C, this cluster contained several genes involved in cell wall-related processes (S2 Table).

**Fig 3:**
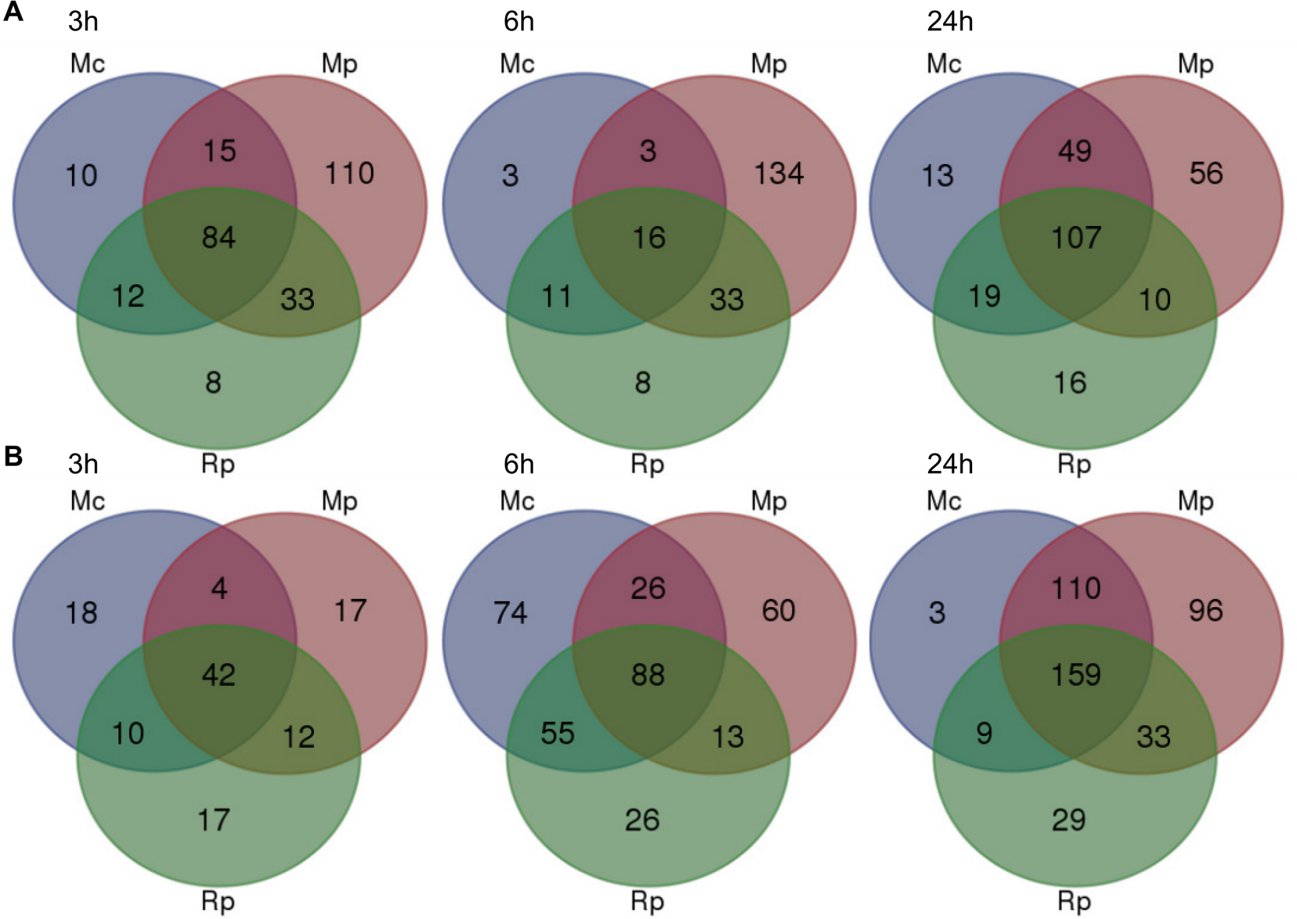
Overlap of *Arabidopsis* differentially expressed genes across different aphid interactions. Venn diagram analyses of 874 differentially expressed as determined by one-way ANOVA (Bonferroni correction, p-value ≤ 0.05) across different aphid interactions and timepoints. (A) Venn diagrams showing the numbers of genes that are down-regulated during different aphid interactions at 3h, 6h and 24h post aphid exposure. (B) Venn diagrams showing the numbers of genes that are up-regulated during different aphid interactions at 3h, 6h and 24h post aphid exposure. Mc indicates *Myzus cerasi*, Mp indicates *M*. *persicae* and Rp indicates *Rhopalosiphum padi*.

We used pairwise analyses of the set of 874 genes to identify down- and up-regulated genes per aphid species treatment per timepoint as compared to the no-aphid control (Fig 3A and 3B). The number of genes down-regulated during the host interaction with *M*. *persicae* was higher than the number of genes down-regulated during the poor-host and non-host interactions, especially at the 3h and 6h timepoint (Fig 3A, S3 Table). Most of the genes significantly down-regulated during the *M*. *cerasi* and *R*. *padi* interactions were also down-regulated during interaction with *M*. *persicae* pointing to overlap in gene regulation taking place during the different types of interactions (Fig 3A, S3 Table). GO analyses of genes significantly down-regulated during all interactions revealed an overrepresentation of genes related to abiotic and biotic stress, such as those encoding small heat shock proteins (SHSPs) or proteins interacting with SHSP at the 3h timepoint. (S4 and S5 Tables). More diverse functions were found for genes commonly down-regulated at the 24h timepoint, with an overrepresentation of genes predicted to function in transcriptional processes.

We assessed whether the gene sets specifically down-regulated during the interaction with *M*. *persicae* showed a similar direction of regulation during the interactions with *M*. *cerasi* and *R*. *padi* by applying a log2 fold change = 0.2 cut off. This showed that at 3h and 24h timepoints 50% (55/110) and just over 70% (41/56) of genes, respectively, showed consistent repression of gene expression for all interactions (S3 Table). When taking into account only the *M*. *persicae* and *R*. *padi* interactions these percentages increased to around 75% (82/110) for the 3h timepoint. However, for the 6h timepoint we only found around 20% (29/134) of *M*. *persicae* down-regulated genes to show consistent changes across all interactions, and this percentage increased to just over 65% when taking into account the *M*. *persicae* and *R*. *padi* data only (S3 Table). Functional predictions suggest that many of the genes significantly down-regulated only during the *M*. *persicae* interaction at both 3h and 6h are involved in ROS metabolism, but also in metabolic processes, including those related to glucosinolate biosynthesis (S3 and S6 Tables).

When assessing genes up-regulated across interactions we also found overlap in gene sets (Fig 3B, S3 Table). Some of the genes commonly up-regulated at 3h were predicted to be related to cell wall functions and growth, while those at the 6h and 24h timepoints were mainly predicted to be involved in transcriptional processes and stress-related responses, respectively (S3 and S6 Tables). However, despite this overlap we also found that more genes were significantly affected during the interaction with *M*. *persicae* at 24h post aphid challenge when compared to the other aphid interactions (Fig 3B, S3 Table). In addition, there was more overlap in genes differentially up-regulated during both the *M*. *persicae* and *M*. *cerasi* interactions at this timepoint than during both the *M*. *persicae* and *R*. *padi* interactions (Fig 3B).

We then looked whether the genes specifically up-regulated during the *M*. *persicae* interaction where affected in the same direction during other interactions. Using a log2 fold change = 0.2 value cut-off, we found that for the 3h and 24h timepoint nearly 45% (8/17) and just over 70% (69/96), respectively, were up-regulated across interactions (S3 Table). When comparing the *M*. *persicae* and *R*. *padi* data only, these percentages increased to around 75% (13/17) for the 3h timepoint (S3 Table). In contrast, at the 6h timepoint only 40% (24/60) of the genes were affected in an upward direction across interactions (S3 Table). Functional predictions showed that those genes significantly and specifically up-regulated by *M*. *persicae* at 6h were likely involved in plant abiotic stress responses, hormone signalling, or metabolic processes (S3 and S6 Tables). Overall these data suggest that *Arabidopsis* responses to *M*. *persicae* are stronger than the responses to the other species, but also that some gene sets may be specifically down-regulated during the host interaction, especially at early timepoints.

### *Arabidopsis* transcriptome analyses identified genes with different and opposite gene expression changes during interactions with *M*. *persicae*, *M*. *cerasi*, and *R*. *padi*

Global analyses revealed that there were sets of genes with significant differential expression in host but not non-host interactions and vice versa, as well as genes showing opposite gene expression changes in different interactions (S1 and S3 Tables). To further look into these gene sets, we analyzed in more detail the one-way ANOVA results of the 874 genes differentially expressed in our experimental set-up. We compared gene sets differentially up- or down-regulated or unaffected during interaction with *M*. *persicae* with similar genes sets for the *M*. *cerasi* or R. *padi* interactions to look for genes specifically differentially expressed during either the host, poor-host or non-host interaction and also applied volcano plot filtering. A total of 96 genes showed either opposite gene expression patterns when comparing two different interactions to the no-aphid control or were only differentially expressed in host, poor-host or non-host interaction (S2–S4 Figs and S7–S9 Tables). Functional predictions of these 96 genes showed an overrepresentation of genes involved in metabolic processes, including glucosinolate biosynthesis, and ROS production (S10 Table). Some genes were specifically up- or down-regulated in only one or two of the interactions (S7 Table). For example, two genes predicted to encode glucosinolate S-oxygenases were down-regulated during interactions with *M*. *persicae* and *R*. *padi* at 6h (only significantly in the *M*. *persicae* interaction), but were not affected during interaction with *M*. *cerasi* at this timepoint. This suggests some plant defence responses may be differentially regulated during interactions with the different aphid species.

**Fig 4:**
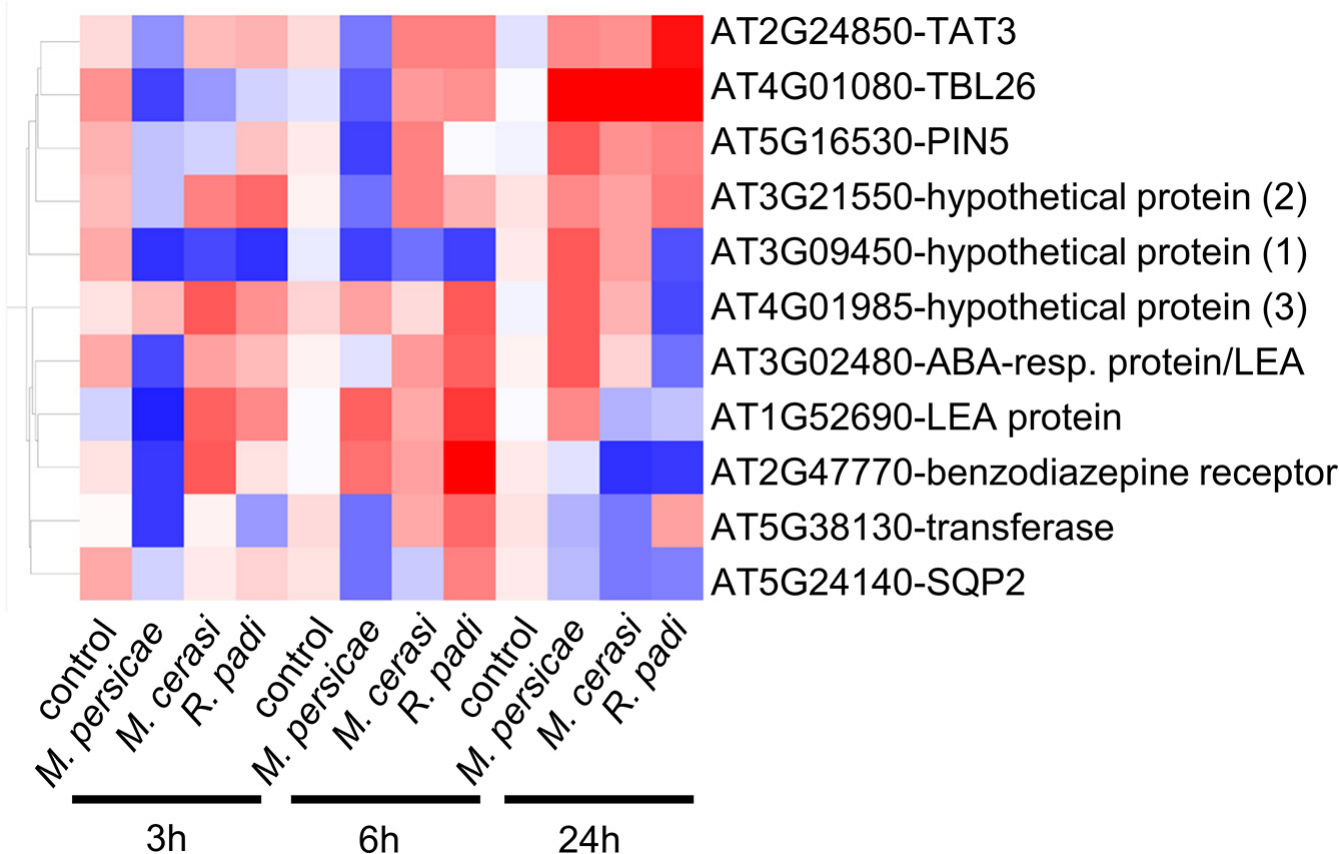
Expression profile of *Arabidopsis* genes showing opposite gene expression changes during aphid host and non-host interactions. Using volcano plot analyses (fold change ≥ 2.0, p-value ≤ 0.05) in GeneSpring, we identified 11 genes with statistically significant opposite gene expression changes during different aphid interactions. Hierarchical gene tree cluster analysis in GeneSpring generated an overview of the expression changes of these 11 genes 3h, 6h and 24h after aphid exposure. Low gene expression levels are indicated by blue color and high gene expression levels are indicated by red color.

The list of 96 genes with different gene expression profiles included a total of 11 genes identified by volcano plot filtering, including genes encoding LEA (Late Embryogenesis Abundant) proteins (AT3G02480, AT1G52690), a transferase (AT5G38130), an oxidoreductase (AT5G24140), PIN5 (PIN-formed 5) (AT5G16530), a benzodiazepine-related receptor (AT2G47770), TBL26 (Trichome Birefringence-Like protein) (AT4G01080), and TAT3 (tyrosine aminotransferase 3) (AT2G24850) (Fig 4). To confirm gene expression profiles we performed quantitative RT-PCR for these 11 genes. Although expression profiles across all timepoints were confirmed for most genes, for three genes (*hypothetical gene 1*, *2* and *3*) we did not confirm opposite gene expression profiles by qRT-PCR (S5 Fig). However, for *hypothetical genes 2* and *3* we observed different gene expression profiles across interactions, with expression being affected specifically during the *M*. *persicae* but not *R*. *padi* interaction at the 6h and 24h timepoint, respectively (S5 Fig). We also performed qRT-PCR analyses on 3 genes (*WRKY38*, *VSP1* (*Vegetative Storage Protein 1*), and a *Gln-amidotransferase*) that were selected based on gene expression profiles during host versus non-host interactions (S7 Table), and two genes (*hypothetical gene 4 and MIOX4* (*Myo-Inositol Oxygenase 4*) that showed interesting patterns of gene expression, but were not identified by our statistical analyses as differentially expressed. Overall, qRT-PCR results were in line with the microarray analyses for these additional 5 genes (S5 Fig). For *MIOX*4 qRT-PCR results revealed more pronounced gene expression differences across interactions than found by microarray analyses, especially for the 24h timepoint (S5 Fig). By applying stringent statistical analyses to select genes with different gene expression profiles we therefore most likely missed some genes of interest.

### Aphid performance assays on *Arabidopsis* knock-out lines reveal genes contributing to host susceptibility to *M*. *persicae* and non-host defences against *R*. *padi*

**Fig 5:**
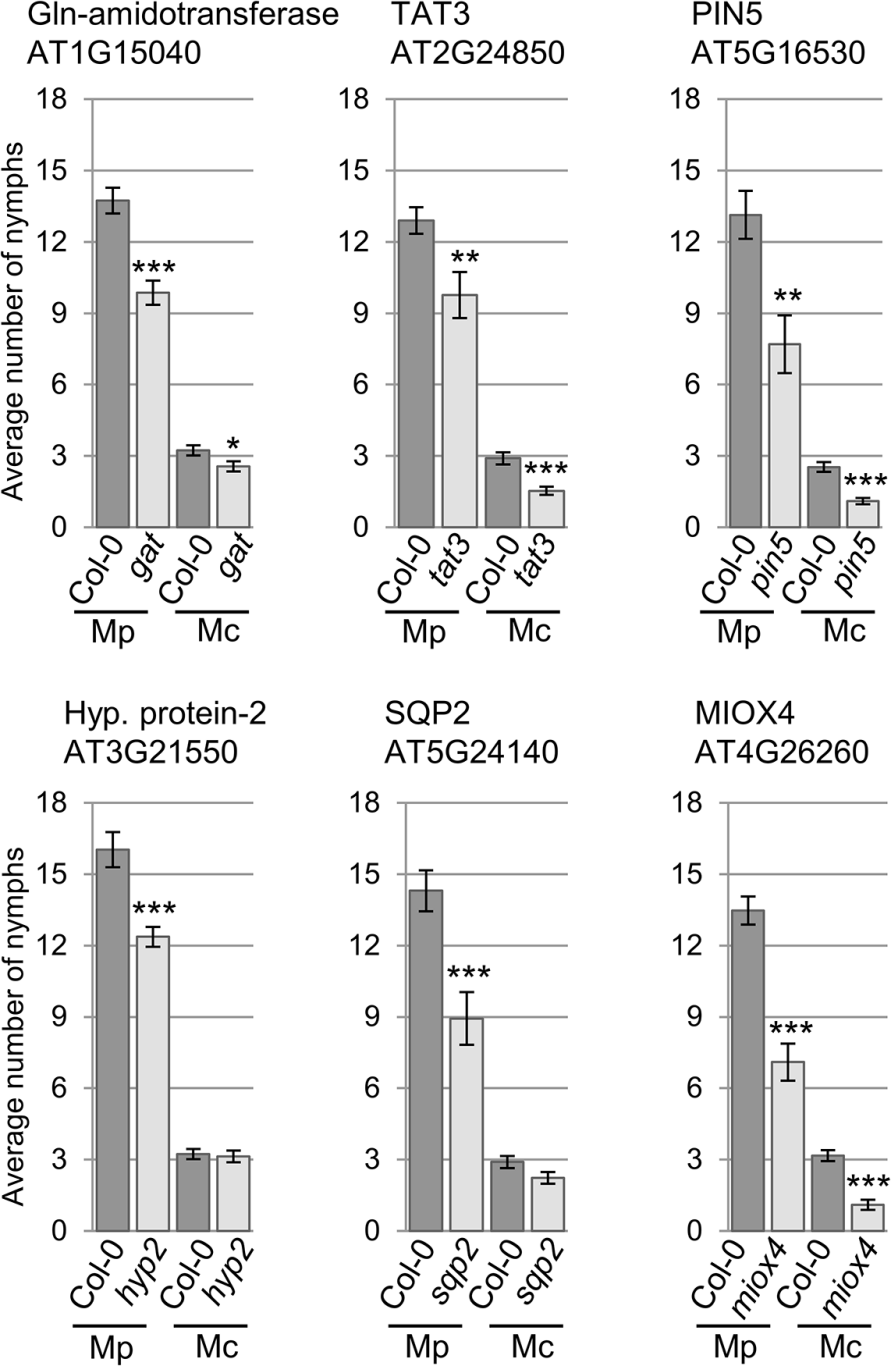
*Arabidopsis* knock-out mutants show altered susceptibility to *Myzus persicae* and *Myzus cerasi*. Four-week old plants were exposed to two adult aphids and nymph production was counted after 10 days. Average nymph production was calculated from three independent replicated experiments, with 10 plants per replicate per treatment. Error bars indicate standard error. The two-tailed Student's *t*-test was used for statistical analyses (*** indicates p-value < 0.001, ** indicates p-value < 0.01, * indicates p-value < 0.05). Mc indicates *Myzus cerasi*, Mp indicates *M*. *persicae*.

**Fig 6:**
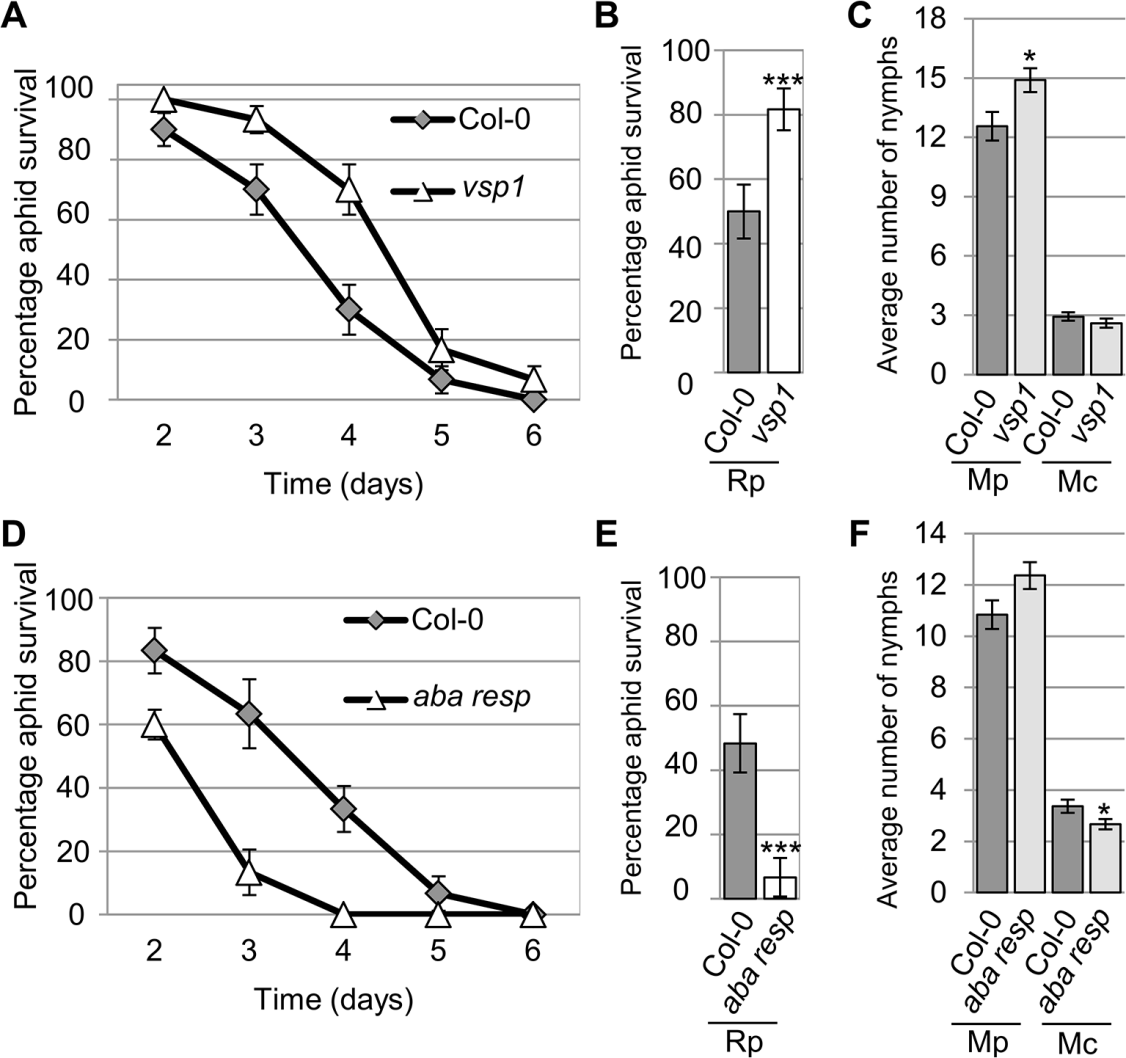
*Arabidopsis* knock-out mutants show altered susceptibility to *Myzus persicae*, *Myzus cerasi* and *Rhopalosiphum padi*. (A) Graph showing *R*. *padi* aphid survival on the control (Col-0) and a *vsp1* mutant line over 6 days. Five adult aphids were placed on four-week old plants and survival was monitored the following 6 days. Three independent biological replicates were carried out, with 10 plants per replicate. Error bars indicate standard error. (B) Graph shows the percentage of *R*. *padi* survival between day 3 and 4 on a *vsp1* mutant line and Col-0 wild-type plants. Data is from the same experiment as described in (A). The two-tailed Student's *t*-test was used for statistical analyses (*** indicates p-value<0.001). Error bars indicate standard error. (C) *M*. *persicae* and *M*. *cerasi* performance on an *Arabidopsis vsp1* knock-out line and Col-0 wild-type plants. Four-week old plants were exposed to two adult aphids and nymph production was counted after 10 days. Average nymph was calculated from three independent replicated experiments, with 10 plants per replicate per treatment. The two-tailed Student's *t*-test was used for statistical analyses (* indicates p-value<0.05). (D) Graph showing *R*. *padi* aphid survival on the control (Col-0) and a mutant line for an *Arabidopsis* gene predicted to encode an ABA-responsive protein over 6 days. Same experimental set-up and analyses as described in A. Error bars indicate standard error. (E) Graph showing the percentage of R. padi survival between day 3 and 4 on a mutant line for an *Arabidopsis* gene predicted to encode an ABA-responsive protein and Col-0 wild-type plants. Data are from the same experiment as described in D. The two-tailed Student's *t*-test was used for statistical analyses (*** indicates p<0.001). Error bars indicate standard error. (F) *M*. *persicae* and *M*. *cerasi* performance on a mutant line for an *Arabidopsis* gene predicted to encode an ABA-responsive protein and Col-0 wild-type plants. Same experimental set-up as described in (C). The two-tailed Student's *t*-test was used for statistical analyses (* indicates p-value < 0.05).

We were interested in investigating whether genes with different gene expression changes during the different type of aphid interactions contributed to host and non-host plant defences against the aphids. Therefore, we selected knock-out lines for 8 of the 11 genes identified by volcano plot filtering, as well as the 5 genes selected based on their gene expression profiles during host versus non-host interactions (S11 Table). This set included a mutant line for *hypothetical gene 1*, for which we were unable to verify differential gene expression across treatments by qRT-PCR. Upon confirming T-DNA or transposon insertions (S6 Fig), we subjected these lines to aphid performance assays. For *M*. *cerasi* and *M*. *persicae* we assessed aphid performance by measuring nymph production over 10 days, whereas for *R*. *padi*, which does not reproduce on *Arabidopsis*, we measured adult aphid survival over 6 days. The overall reproduction of *M*. *cerasi* was very low in our experiments, reflective of poor-host suitability to this species (Figs 5 and 6). A slight reduction in performance was observed for *M*. *cerasi* on several knock-out lines (*Gln*-*amidotransferase*, *pin5*, *miox4*) (Fig 5). Interestingly, *M*. *persicae* showed a significant reduction in reproduction on the *pin5*, *miox4*, *sqp2*, *hypothetical gene 2*, *tat3* and *Gln-amidotransferase* lines indicating that the regulation of these genes is important for virulence in host interactions (Fig 5). Possibly these genes encode aphid susceptibility factors or aphids require a tight regulation of the processes these genes are involved in. Non-host resistance to *R*. *padi* was not affected in these mutants (S7 Fig).

Aphid survival assays with *R*. *padi* identified several lines affected in non-host resistance to this aphid species. More specifically, we observed increased survival of *R*. *padi* on the *vsp1* mutant, indicating VSP1 (vegetative storage protein 1) contributes to non-host resistance against this aphid (Fig 6A and 6B). While on Col-0 plants *R*. *padi* survival was around 50% between 3 and 4 days of the assays, on the *vsp1* mutant survival was around 80%, which was significantly higher. Aphids did not survive beyond 6 days on either the wild-type or *vsp1* mutant plants. The *vsp1* line showed a significant increase in progeny of *M*. *persicae*, indicating that VSP1 also contributes to host defences against this aphid species (Fig 6C). Another interesting observation was that *R*. *padi* showed decreased survival on a mutant affected in the expression of an ABA-responsive gene, which is a member of the *LEA* gene family (Fig 6D and 6E). Between 3 and 4 days of the assays, aphid survival on wild-type plants was around 50%, but reduced to about 7% on the mutant. We did not observe any difference in susceptibility of this mutant to *M*. *persicae* as compared to the Col-0 wild-type (Fig 6F). This may indicate that this *LEA* gene negatively regulates plant defences to specific aphid species. Remaining lines were not affected in susceptibility or non-host resistance (S8 Fig). Our results show that several of the genes identified by their differential gene expression profiles during the host, poor-host, and non-host interactions play an important role during plant-aphid interactions.

### ROS production in *Arabidopsis* is activated upon interaction with *M*. *persicae*, *M*. *cerasi*, and *R*. *padi*

**Fig 7:**
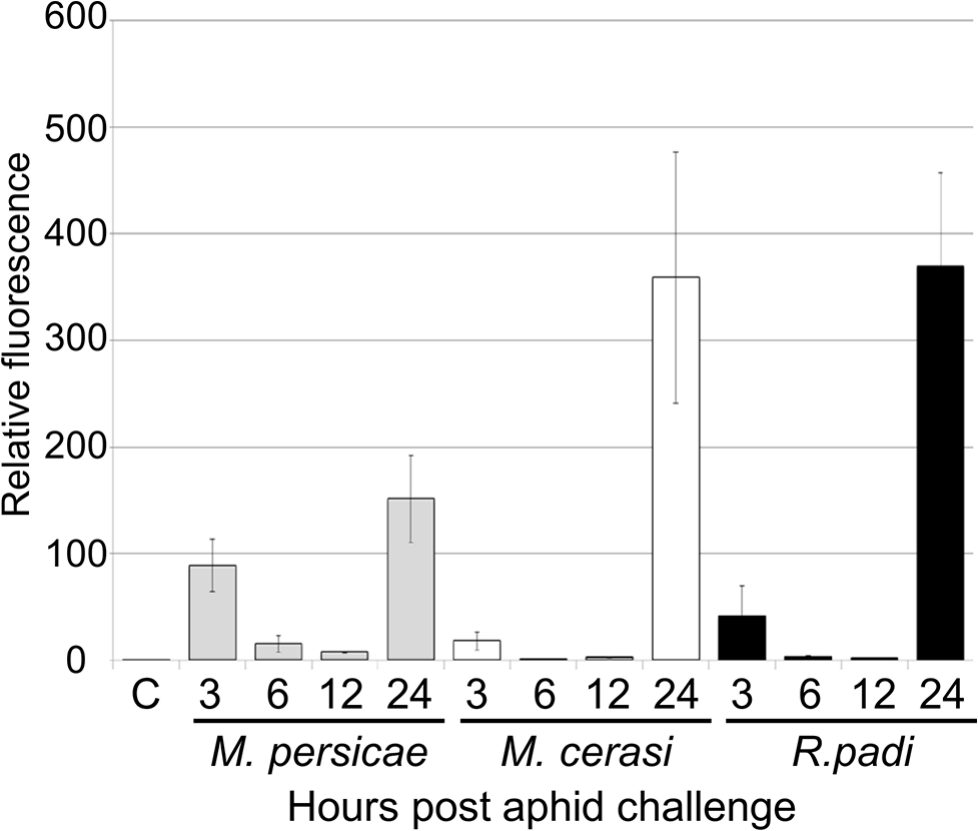
ROS levels during host and non-host interactions with aphids. Leaves exposed to different aphid species were incubated with the dye DCFH-DA (dichlorodihydro-fluorescein diacetate) to compare reactive oxygen species (ROS) levels during host and non-host interactions. Images were taken 3, 6, 12 and 24 hours after aphid exposure using a laser confocal microscope and processed in ImageJ to generate graph bars representing relative fluorescence to the control treatment (no aphids). Graph indicates fluorescence ratios of leaf samples exposed to aphids compared to a no-aphid control (C). Average relative ratios are based on 5 different leaf samples per treatment. Three independent replicated experiments, with 5 leaf samples per treatment per replicate, were performed with similar results. Graph shows results of one of the two replicates. The additional replicate is shown in S9 Fig.

Our transcriptome analyses revealed that genes involved in ROS metabolism were repressed more strongly upon interaction with the aphid species *M*. *persicae* as compared to the poor-host and non-host interactions. To further investigate the role of ROS production in plant-aphid interactions, we used the dye DCFH-DA to assess ROS accumulation over a 24-hour timecourse experiment. Leaves were challenged with five *R*. *padi*, *M*. *cerasi* and *M*. *persicae* aphids, or no aphids (control) and subjected to staining after 0, 3, 6, 12 and 24 hours. Leaves were treated with DCFH-DA and analyzed by confocal microscopy to measure the accumulation of ROS. Across repeated experiments we observed an accumulation of fluorescence, indicative of ROS production, peaking at 3 and 24 hours post aphid challenge in host, but mainly at 24 hours in non-host or poor-host interactions (Figs 7, S9). Moreover, at 24 hours ROS production was more pronounced in poor-host and non-host interactions with *M*. *cerasi* and *R*. *padi*. To determine whether accumulation was possibly the result of an active process or recognition of the aphid exoskeleton, we challenged leaves with aphid moults, containing chitin, and assessed ROS production at 24 hours post challenge. We did not observe an increased accumulation of ROS in these experiments (S9 Fig), suggesting that activation of oxidative responses is not due to recognition of chitin at the leaf surface but rather requires aphid feeding and/or probing to take place.

### NAPDH oxidase AtRbohF, but not AtRbohD, contributes to *Arabidopsis* host and non-host defences against *M*. *persicae*, *M*. *cerasi*, and *R*. *padi*

**Fig 8:**
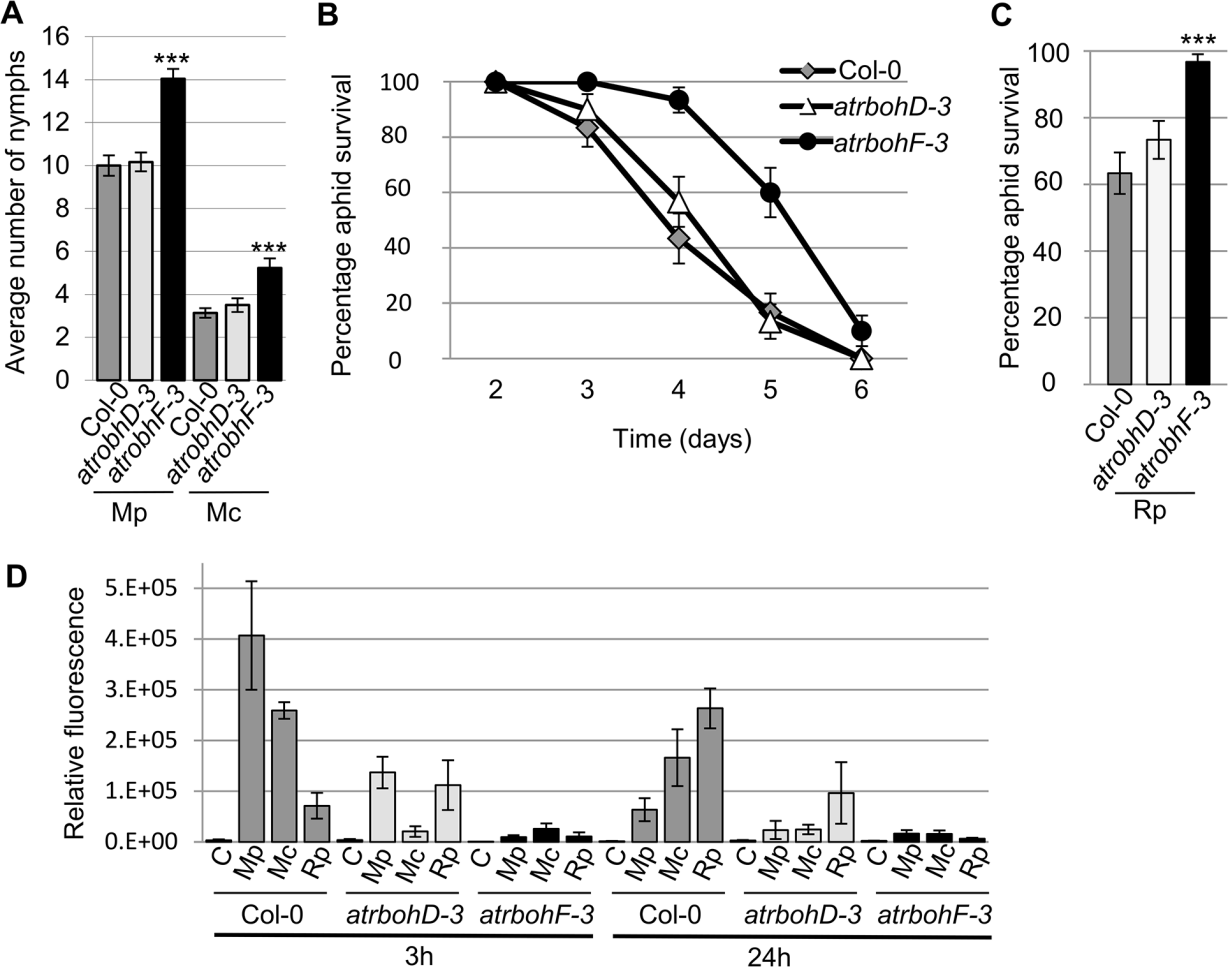
*Arabidopsis atrbohD-3* and *atrbohF-3* knock-out mutants show reduced non-host resistance and enhanced susceptibility to aphids. (A) *Myzus persicae* (Mp) *and M*. *cerasi* (Mc) performance on *Arabidopsis atrbohD-3* and *atrbohF-3* knock-out lines. Four-week old plants were exposed to two adult aphids and nymph production was counted after 10 days. Average nymph production for *M*. *persicae* and *M*. *cerasi* was calculated from three independent replicated experiments, with 10 plants per replicate per treatment. (B) Graph shows *R*. *padi* aphid survival on control (Col-0) and *Arabidopsis atrbohD-3* and *atrbohF-3* knock-out lines over 6 days. Three independent biological replicates were carried out, with 10 plants per replicate. Error bars indicate standard error. (C) *Rhopalosiphum padi* (Rp) survival on *Arabidopsis atrbohD-3* and *atrbohF-3* mutants and Col-0 wild-type plants between 3 and 4 days of the experiment. The two-tailed Student's *t*-test was used for statistical analyses (*** indicates p-value<0.001). Error bars indicate standard error. (D) ROS levels in *atrbohD-3* and *atrbohF-3* mutants during host and non-host interactions with aphids. Images were taken 3 and 24 hours after aphid exposure using a laser confocal microscope and processed in ImageJ to generate graph bars representing relative fluorescence to the control treatment (no aphids). Graph indicates fluorescence the relative fluorescence of leaf samples exposed to aphids compared to a no aphid control (indicated by C). Average relative ratios are based on 5 different leaf samples per treatment. Three independent replicated experiments, with 5 leaf samples per treatment per replicate, were performed with similar results. Graph shows results of one of the three replicates. Additional replicates are shown in S10 Fig.

The production of ROS is dependent on NADPH oxidases. Two of these, AtRbohD and AtRbohF were previously shown to be involved in plant responses to biotic stress [20]. We challenged *atrbohD-3* and *atrbohF-3* knockout lines with *R*. *padi*, *M*. *cerasi* and *M*. *persicae* and monitored aphid population size over 10 days to determine if *AtRbohD* and *AtRbohF* contribute to plant defences in host and non-host interactions. Both *M*. *cerasi* (poor-host interaction) and *M*. *persicae* (host interaction) showed an increase in population size on the *atrbohF-3*, but not the *atrbohD-3* line, compared to the wild-type control (Fig 8A). As expected *R*. *padi* did not reproduce on either the wild type or knockout lines. However, when assessing adult survival we found that 90% of aphids were still alive between day 3 and 4 on the *atrbohF-3* line as opposed to 60% on the wild-type Col-0 plants (Fig 8B and 8C). No difference in aphid survival was observed on the *atrbohD-3* line. These results indicate that *AtRbohF*, but not *AtRbohD*, contributes to plant defences in the different types of aphid interactions and that this NADPH oxidase contributes to non-host resistance against aphids.

To determine whether the *atrboh* mutants were able to generate ROS during aphid interactions, we performed DCFH-DA staining of leaves at 3h and 24h post aphid challenge. Although ROS levels were strongly reduced in both the *atrbohD-3* and *atrbohF-3* mutants for all interactions, the amount of ROS was more strongly reduced in the *atrbohF-3* mutant than in the *atrbohD-3* mutant, especially at the 3h timepoint (Figs 8D, S10). These data point to different roles of NADPH-oxidases in the production of ROS during plant-aphid interactions.

## Discussion

Here, we provide novel insights into plant responses during interaction with three different aphid species and identified several genes involved in host susceptibility or immunity to *M*. *persicae* as well as non-host resistance against *R*. *padi*.

Gene expression analyses across timepoints and interactions showed high levels of overlap in transcriptional responses but also revealed genes that were differentially regulated. There are several possibilities that may explain the relatively small number of genes differentially affected during the different aphid interactions. It is possible that 1) only a relatively small number of plant genes affect host range, and/or 2) that changes at the protein level rather than transcript level are important in host versus non-host defences against aphids, and/or that 3) in addition to a set of plants genes, aphid genes play a key role in determining aphid host range. In addition, the stronger repression of plant transcriptional responses at early timepoints upon interaction with *M*. *persicae* as compared to the other interactions could reflect host transcriptional reprogramming by this aphid species to suppress host defences and enable infestation. It is likely that aphid effectors secreted with saliva into host plants not only target protein functions, but also manipulate host processes by targeting regulation of gene expression.

Our microarray analyses provided limited evidence for activation of PAMP-responsive genes during the different plant-aphid interactions. We assessed expression profiles of several genes that are activated upon PAMP treatment. This showed that *RLK1* (receptor-like kinase 1) and *RLK5* (receptor-like kinase 5) were both down-regulated at 3h, but showed similar profiles across interactions (S1 Table). Also, *YLS9* (AT2G35980) was highly upregulated during all interactions at 24h, while *PH1* (AT1G35140) was down-regulated at 3h and 24h. *FRK1*, *CYP81F2* and *WRKY22* were not significantly affected upon aphid challenge. Overall, we did not observe any specific gene expression profiles of PAMP-responsive genes for non-host versus host interactions. Possibly, other unknown signaling pathways may be involved in the recognition of aphids or aphid-associated organisms.

Arabidopsis mutant analyses revealed several genes that are important for aphid virulence and contribute to susceptibility to *M*. *persicae* and/or *M*. *cerasi*. PIN5 is an endoplasmic reticulum (ER)-localized transporter involved in regulating auxin influx into this organelle to regulate auxin homeostasis [27]. The role of auxin signaling in plant-aphid interactions is unknown, but possibly tight regulation of auxin is important for aphid virulence. Also, the UPR (untranslated protein response), which is triggered by ER stress, is reduced in *pin5* mutants [28]. Possibly this response is important for activation of defences against aphids and dealing with biotic stress. One of the other genes, *MIOX4*, is highly expressed in *Arabidopsis* flowering tissues, and is potentially involved in the synthesis of cell wall polysaccharides [29]. This enzyme can convert myo-inositol to D-glucuronic acid, which is a major precursor for cell wall polysaccharides. *MIOX4* is also highly upregulated in syncytia during infection by the plant parasitic nematode *Heterodera schachtii* [30]. A *miox* quadruple knockout mutant (*Δmiox1/2/4/5*), showed reduced susceptibility to nematodes. However, cell wall composition seems unaffected in this mutant possibly due to up-regulation of another polysaccharide synthesis pathway [31] and it was suggested that an increase in metabolites, including galactinol, may be responsible for the effects on nematode infection [32]. Whether metabolites are similarly affected in the *miox4* mutant, remains to be investigated.

*VSP1*, which contributed to both host defences against *M*. *persicae* and non-host resistance to *R*. *padi*, encodes a putative acid phophatase. Although *VSP1* transcripts are jasmonate-inducible [33], the observed down- and up-regulation during the host and non-host interactions, respectively, is not necessarily linked to repression or activation of JA-signaling. For example, other JA-responsive genes, like *LOX3*, and *SEN1* show induction across interactions (S1 Table). The JA-responsive marker *PDF1*.*2* was also induced across all aphid interactions, but due to replicate-to-replicate variation this induction was not statistically significant (https://www.ebi.ac.uk/arrayexpress/E-MTAB-3223). A close relative of *VSP1*, *VSP2*, showed a similar gene expression profile to that of *VSP1*, despite not being identified as differentially expressed between aphid interactions using volcano filtering (S1 Table). Interestingly, VSP2 recombinant protein has anti-insect activity when added to an artificial diet provided to several insect species [34]. It is therefore possible that VSP1 has a direct and general anti-insect activity. Alternatively, VSP1 may have broader a role in plant defences, which could explain why its expression is induced by biotic and abiotic stress [33], [35].

Two *LEA* genes were differently regulated across interactions, one of which increased non-host resistance to *R*. *padi*. The LEA family contains many different members that are likely involved in cell stress tolerance and osmoregulation [36], [37], [38], [39]. Some of these proteins have been implicated in biotic stress responses [40]. More specifically, overexpression of *ZmLEA3* increased the hypersensitive response triggered by avirulent *P*. *syringae pv tomato* in transgenic tobacco. *AtLEA5* also was implicated in biotic stress as its overexpression in *Arabidopsis* reduced virulence of the fungal pathogen *Botrytis cinerea* as well as virulent *P*. *syringae pv tomato* [41]. Interestingly, this LEA member is involved in oxidative stress tolerance [42]. Also, NDR1 (non-race-specific disease resistance 1), which is important for signaling during ETI triggered by R proteins, shares homology to a member of the LEA protein family, LEA14 [43]. Although the role of members of the LEA family in biotic and abiotic stress is largely unknown, our work shows some LEA proteins may be involved in regulating non-host resistance to aphids.

*M*. *cerasi* performance was less affected than *M*. *persicae* or *R*. *padi* on select *Arabidopsis* mutants. However, *M*. *cerasi* reproduction rates on *Arabidopsis* were low due to poor host suitability, resulting in small numbers of nymphs being produced. It is possible that different molecular mechanisms are important for *M*. *cerasi* virulence than for *M*. *persicae* or *R*. *padi*.

Production of reactive oxygen species (ROS) is one of the early plant defence responses upon interaction with pathogens [44]. Here we used DCFH-DA as a tool for visualizing the accumulation of ROS and provide us insight into the overall accumulation of ROS upon aphid challenge. One limitation of this approach is that it does not allow to distinguish between intra- and extracellular pools of ROS [45]. However, plasma membrane NADPH-oxidases are involved in the apoplastic production of ROS. Therefore, our observations that ROS production upon aphid interaction was (partially) dependent on NADPH-oxidases, and that one of these NADPH-oxidases contributed to plant defences against aphids suggest that at least the production of apoplastic ROS is involved.

The very early ROS response (3h post challenge) observed mainly and consistently in the presence of *M*. *persicae* (host interaction), may be due to early recognition events. Herbivore feeding can cause mechanical damage resulting in an increased accumulation of ROS [46]. However, aphids are considered stealthy herbivores that cause little damage to plant cells. Despite this we did notice significant damage to epidermal cells when assessing aphid probes (Figs 1A, S1), which could be responsible for activation of wound-like defences. Alternatively, aphid saliva, containing elicitors, could be responsible for the activation of oxidative stress at early timepoints. In either case, the difference in ROS production at the very early timepoint between the host and non-host interactions could reflect differences in damage caused by the aphids due to probing, or differences in saliva or elicitor delivery. The more pronounced ROS response in the non-host and poor-host interactions at the 24h timepoint may reflect a stronger activation of plant defences. Indeed, the enhanced susceptibility of the *atrbohF-3* mutant to *M*. *persicae*, but importantly the reduced level of non-host resistance of this mutant to *R*. *padi*, suggest that the *Arabidopsis* oxidative response negatively impacts aphid performance regardless of the aphid species it is interacting with. However, ROS accumulation at 24h post aphid challenge was reduced in the *atrbohF-3* as well as the *atrbohD-3* mutant, suggesting that AtRbohF contributes to host and non-host defences through a mechanism other than ROS production, or that AtRbohF and AtRbohD are involved in different oxidative responses. Interestingly, Chaouch et al. [47] showed the AtRbohF is involved in the intracellular oxidative responses upon pathogen attack to regulate defences and metabolic process, and identified AtRbohF specific functions. Both SA and camalexin accumulation are reduced in *atrbohF-3*, but not *atrbohD-3*, mutants upon challenge with the plant pathogen *P*. *syringae pv tomato* DC3000 [47]. *Arabidopsis* mutants that are unable to produce camalexin are more susceptible to *M*. *persicae* [10], pointing to a role of camalexin in plant defences against aphids. It is possible that differences in camalexin levels in the *atrbohF* mutant affect defences in both host and non-host interactions. SA has also been implicated in plant-aphid interactions, but the exact role of SA-mediated defences remains unclear. It has been speculated that the activation of SA-signaling pathways by aphids counters the activation of JA-dependent defence responses that are effective against aphids [48]. However, high levels of SA-signaling do not necessarily correspond with increased susceptibility to aphids [49].

We did not observe a change in aphid virulence on the *atrbohD-3* knockout mutant, which is in contrast to what has been reported by Miller et al. [21], where *M*. *persicae* was more virulent on this particular mutant. Although we used the same available *atrbohD-3* mutant line in our experimental set-up as Miller et al [21], plants were grown under short-day conditions rather than under constant light. Not only can constant light conditions impact aphid reproduction [50], but also plant physiology [51]. We did, however, observe a reduction in ROS production in the *atrbohD-3* mutant upon aphid challenge, in line with a role of AtRbohD in ROS production upon treatment with aphid-derived extract [12]. However, ROS production has been reported in *atrbohD-3* and *atrbohF-3* mutants indicating other genes may contribute to oxidative responses [52].

We used three different aphid species in our study, representing host-, poor-host and non-host interactions. Although our work is an important step forward in characterizing and comparing host and non-host plant responses to aphids, it will be important to investigate whether our observations extend to similar interactions with other aphid species. For example, Arabidopsis *PEN1* (*PENETRATION1*) and *PEN2* differ in their contribution to non-host resistance against a range of fungal and oomycete pathogens with *PEN1* showing a higher level of specificity [53]. Further characterization of the plant genes identified here is needed to determine whether they contribute to host and non-host interactions on a broader scale.

With aphids being a major economic pest it is essential to understand the molecular basis of host susceptibility and host range. Understanding how plants respond to different aphid species that differ in their ability to infest plant species, and identifying the genes and signaling pathways involved, is essential for the development of novel and durable aphid control in crop plants. Overall our work contributes to a better understanding of the molecular mechanisms underlying host and non-host interactions with aphids. Further characterization of plant genes important for host susceptibility or non-host resistance will be needed to investigate whether their functions extend to other plant-aphid interactions and to reveal the plant cellular processes involved in determining aphid virulence and host range.

## Methods

### Insect rearing

The aphids used in this study are *M*. *persicae* (genotype O, kindly provided by Dr. B Fenton), *M*. *cerasi* (collected from cherry trees in Dundee, UK) and *R*. *padi* (kindly provided by Dr. B Fenton). *M*. *persicae* aphids were reared on oilseed rape (*Brassica napus*), *M*. *cerasi* on American land cress (*Barbarea verna*) and *R*. *padi* on barley (*Hordeum vulgare L*.). The insects were maintained in cages in controlled conditions at 20°C under 16h of light.

### Aphid colonization and probing

Aphids were age-synchronized on host plants. For *M*. *persicae* the host was *B*. *napus*, for *M*. *cerasi* American land cress and for *R*. *padi* this was barley. Two 8-day old adults were moved to four-week old *Arabidopsis* plants. Plants were individually caged and aphids were counted 14 days later. For colonization of cress by *M*. *cerasi* a similar experiment was performed at a later timepoint under the same conditions. Probing was assessed using confocal laser microscopy using an excitation at 488 nm and emission ranges of 500–530 nm (793 of master gain) and 650–690 nm (590 of master gain).

### Microarray experimental design and analyses

*Arabidopsis thaliana* Col-0 plants were grown under short day conditions (8h light (±80μmoles.m^-2^.s^-1^ /16h dark) at 22°C (light)/ 20°C (dark), 70% humidity. Plants were sown on Levington's M2 compost with 4 mm grit (8:1). Individual four-week old plants were challenged with 25 mixed-age apterous aphids and enclosed in a mesh-covered cylindrical cage. Control plants (non-infested), were placed in cages in parallel. We used 15 plants per aphid treatment, per timepoint, for each replicate and performed three biological replicates. For each aphid treatment, and per replicate, all above ground tissues from 15 plants were collected and pooled after 3h, 6h or 24h and flash-frozen in liquid nitrogen. Samples were ground in liquid nitrogen and total RNA was extracted using TRIzol Reagent (Invitrogen, Life Technologies, Carlsbad, CA, USA) as described by the manufacturer.

The quality of RNA was assessed using the Agilent Bioanalyzer. For microarray analyses, slides were hybridized with three biological replicates per aphid treatment per timepoint. The microarray experimental design and dataset can be accessed at ArrayExpress (https://www.ebi.ac.uk/arrayexpress/E-MTAB-3223; accession #E-MTAB-3223). The Low Input Quick Amp Labeling kit (Agilent Technologies, Santa-Clara, CA, USA) was used according to the manufacturer’s instructions to amplify and label target RNA. Arabidopsis v4 Gene Expression Microarrays (Agilent Technologies) containing 43,803 probes were used (36 in total). Single-colour hybridization and washing of the slides were performed according to the manufacturer’s protocols (Agilent Technologies; One-Color Microarray-Based Gene Expression Analysis, version 6.5). An Agilent Technologies G2505B scanner was used to scan the hybridized slides at resolution of 5 μm at 532 nm (Cy3).

Data were extracted from each microarray using Feature Extraction software (Agilent Technologies version 10.7.3.1) with default settings and subsequently data were imported into GeneSpring (version 7.3; Agilent Technologies) software for analyses. One-way ANOVA (Bonferroni correction, p-value ≤ 0.05) was used to identify genes differentially expressed across different treatments compared to the non-infestation controls (S3 and S4 Figs, S9 Table). To identify genes with opposing gene expression profiles among treatments, we performed volcano plot filtering (fold change ≥ 2.0, t-test p-value ≤ 0.05) (S2 Fig, S8 Table).

### GO enrichment analyses

We used BioMaps software available on the Virtual Plant web platform, version 1.3, (http://virtualplant.bio.nyu.edu/cgi-bin/vpweb/, [54]) to analyse gene ontologies (GO) and functional annotations from the Munich Information Center for Protein Sequences (MIPS) [55]. For these analyses we selected the TAIR/TIGR (The Arabidopsis Information Resource/ The Institute for Genomic Research) database and applied the Fisher Exact Test (with FDR (False discovery rate) correction, p-value ≤ 0.01).

### Validation of gene expression changes by q-PCR

Real-time qPCR was performed on a Chromo4 System (Bio-Rad, Hercules, CA, U.S.A.) with Opticon 3.1 software, as follows: 95°C for 10 min followed by 44 cycles of 95°C for 15 s and 60°C for 1 min, with the qPCR MasterMix sybR green (Applied Biosystems, Life Technologies, Carlsbad, CA, USA). Primer efficiency (E) was evaluated on a slope of a standard curve generated using a serial dilution (4 dilution points-2 fold dilution) of the mixed sample (E = 10^^(-1/slope)^-1). Each sample reaction was run in triplicate. Cycle threshold (Ct) values were normalized to the average Ct of three housekeeping genes, elongation factor EF1α (AT1G07920-AtEF1α elongation factor), actin 2 (AT3G18780), and ubiquitin 22 (AT5G10790-carboxyl-terminal hydrolase 22). The expression of these three genes was unaffected in our microarray analyses across treatments, and *ACT*2 has been previously used as a reference gene in qPCR experiments on *Arabidopsis* infested with *M*. *persicae*[10]. Expression levels were quantified by the efficiency calibrated method following this equation ratio = E_sample_^ΔCtsample^/ E_calibrator_^ΔCtcalibrator^). Primers used are summarized in S12 Table.

### Leaf staining

*Arabidopsis* leaves were exposed to 5 adult aphids for 24 hours. Aphids were maintained on leaves using mesh covered clip cages. Leaves were harvested and cleared in 70% ethanol for at least 48 hours. For fuchsin staining, cleared leaves were soaked in a fuchsin acid solution (0,035% in acetic acid:water, 1:3V) for 2 minutes at room temperature, mounted on a glass slide in 100% glycerol and analyzed directly using a light microscope directly. For trypan blue staining, leaves were boiled in Trypan blue solution (lactophenol solution/ EtOH 100% 1:1V, Trypan blue 0,02%) for 3 minutes [56], followed by a 15 minutes incubation at room temperature. Samples were cleared in chloral hydrate solution (1g/mL) for 36 hours, washed twice in 50% of glycerol, and analyzed using a light microscope.

### ROS detection

We used the dye 2’,7’-dichlorofluorescein diacetate (DCFH-DA, Sigma–Aldrich, St. Louis, MO, USA) to determine levels of ROS production in *Arabidopsis* leaves using a protocol previously detailed by Mai et al [19]. Detached leaves of 4-weeks old plants, were placed into a 96-wells plate containing 1% water agar. Detached leaves were exposed to 5 aphids for 0 (control), 3, 6, 12 or 24 hours. We used 5 detached leaves per treatment per replicate and performed three replicates for timecourse experiments including the 3 and 24h timepoints only, and two replicates for timecourse experiments including the 3, 6, 12 and 24h timepoints. Leaves were collected and submerged in 300μM DCFH-DA in 50mM potassium phosphate buffer (pH 7.4) and incubated overnight in the dark. Leaves were washed twice with potassium phosphate buffer for at least 1min and analyzed using a Zeiss LSM 710 confocal microscope (Carl Zeiss, Jena, Germany). Images were converted by the LSM Image Browser software, ZEN 2011, Blue edition (Carl Zeiss) into JPEG files and ROS production was quantified with ImageJ. Graphs were generated for each individual biological replicate as relative values varied per replicate. All images, including those converted for ImageJ analyses, have been deposited in DRYAD (dryad.18b29) [57].

### Performance assays on Arabidopsis mutant lines

All mutants used in this study were in the ecotype Columbia background and were obtained from the NASC (Nottingham Arabidopsis Stock Centre). S11 Table summarizes all mutants used in our study. T-DNA or transposon insertions were confirmed by PCR on genomic DNA and in the case of the AtRbohD and AtRbohF mutants also on cDNA. Primers are listed in S12 Table. Plants were grown under short day conditions (8h light/16h dark) at 22°C (light)/ 20°C (dark), 70% humidity. Four-week old plants were challenged with two 8-day old aphids (age-synchronized) for the *M*. *persicae* treatment and with two similar-size adult aphids for *M*. *cerasi* treatments. For *R*. *padi*, plants were challenged with five similar-size apterous aphids. We used 10 plants per treatment per replicate and the experiment was repeated three times. For *M*. *persicae* and *M*. *cerasi* aphid progeny were counted after 10 days. For *R*. *padi*, aphid survival was assessed from the 2^nd^ to 6^th^ day. To compare the survival rates we took the average number of aphids alive on day 3 and day 4 of the experiment. We performed three biological replicates of aphid performance assays. Statistical analyses were performed using two-tailed Student's t-test.

## Supporting Information

S1 Fig Aphid probing during host, poor-host, and nonhost interactions. Fuchsin stain (A) and Trypan blue stain (B) of Arabidopsis leaves exposed to different aphids species. Scale bars 20 μm (A). Scale bars 20mm (B). Fuchsin staining visualized aphid stylet pathways, whereas Trypan blue staining visualized cell death, as indicated by the arrows. (C) Aphid colonization of American cress by *Myzus cerasi*. Graph shows the mean number of nymphs produced after two weeks on cress plants. Error bars indicate standard error. Three independent biological replicates were carried out, with 4 plants per replicate.
(TIF)

S2 Fig Venn diagram repartition of genes identified by volcano plot filtering. Volcano filtering was used to identify gene with opposite gene expression profiles among treatments. Numbers are numbers of genes identified using statistical analyses of aphid treatment per timepoint versus the non-aphid control (p-value<0.05). Mc indicates *Myzus cerasi*, Mp indicates *M*. *persicae* and Rp indicates *Rhopalosiphum padi*.
(TIF)

S3 Fig Venn diagram repartition of genes differentially regulated during *Myzus persicae* and *Myzus cerasi* interactions. Of the 874 genes in total identified as differentially expressed using one-way ANOVA with Bonferroni correction (p-value<0.05), we generated Venn diagrams to perform pairwise comparisons and identify genes differentially expressed across interactions. Mp indicates *Myzus persicae* and Mc indicates *Myzus cerasi*. Data corresponds to S9 Table.
(TIF)

S4 Fig Venn diagram repartition of genes differentially regulated during *Myzus persicae* and *Rhopalosiphum padi* interactions. Of the 874 genes in total identified as differentially expressed using one-way ANOVA with Bonferroni correction (p-value<0.05), we generated Venn diagrams to perform pairwise comparisons and identify genes differentially expressed across interactions. Mp indicates *Myzus persicae* and Rp indicates *Rhopalosiphum padi*. Data corresponds to S9 Table.
(TIF)

S5 Fig Validation of gene expression by RT-qPCR. Gene expression profiles of selected candidate genes were evaluated using RT-qPCR. Intensity values represent the average of the Log2 (ratio = E_sample_^ΔCtsample^/ E_calibrator_^ΔCtcalibrator^ as calibrator, housekeeping genes *ACT*2, *EF1α* and *UBQ22*). Intensity values ± standard deviation were plotted for each gene. Grey bars represent the expression profiles according to the RT-qPCR results. Black bars represent the average expression profiles according to the microarrays results, based on three biological replicates, and error bars indicate the standard error. Red lines and stars indicate the comparisons found to be differential among interactions by statistical tests (volcano plot analyses/or ANOVA) based on the microarray data. The RT-qPCR experiments were performed on the pooled samples of the three biological replicates.
(TIF)

S6 Fig Confirmation of knock-out lines. (A) The expression of *AtRbohD* and *AtRbohF* was evaluated by RT-PCR in cDNA from the *atrbohD-3* and *atrbohF-3* knockout mutants and wild-type Col-0 plants. (B) Transposon insertions were confirmed by PCR on *atrbohD-3* and *atrbohF-3* genomic DNA. (C) T-DNA insertion confirmation by PCR on genomic DNA of all knock-out lines used in this article.
(TIF)

S7 Fig *Arabidopsis* knock-out mutants not affected in their resistance to *Rhopalosiphum padi*. Graphs showing *R*. *padi* aphid survival on the knock-out mutants, and the control (Col-0) over 6 days. Five adult aphids were placed on four-week old plants and survival was monitored the following 6 days. Three independent biological replicates were carried out, with 10 plants per replicate. Error bars indicate standard error
(TIF)

S8 Fig *Arabidopsis* knock-out mutants not affected in their resistance/susceptibility to *Myzus persicae*, *Myzus cerasi* and *Rhopalosiphum padi*. (A) *M*. *persicae* and *M*. *cerasi* performance on *Arabidopsis* knock-out mutants and Col-0 wild-type plants. Four-week old plants were exposed to two adult aphids and nymph production was counted after 10 days. Average nymph production was calculated from three independent replicated experiments, with 10 plants per replicate per treatment. (B) Graph showing *R*. *padi* aphid survival on the knock mutants and the control (Col-0) over 6 days. Five adult aphids were placed on four-week old plants and survival was monitored the following 6 days. Three independent biological replicates were carried out, with 10 plants per replicate. Error bars indicate standard error.
(TIF)

S9 Fig *Arabidopsis* ROS production during plant aphid interactions. Leaves exposed to different aphid species were incubated with the dye DCFH-DA (dichlorodihydro-fluorescein diacetate) to compare ROS levels during host and non-host interactions. (A) Levels of ROS in Arabidopsis detached leaves after *Myzus persicae*, *M*. *cerasi* and *Rhopalosiphum padi* exposure. Five adult aphids were placed on each leaf and leaves were collected after 3, 6, 12 and 24 hours. Images were taken with a laser confocal microscope. Figure shows example images and all 5 images per treatment per timepoint are available in DRYAD (dryad.18b29) (B) Second replicate of the experiment shown in Fig 7. Images were taken using a laser confocal microscope and processed in ImageJ to generate graph bars representing relative fluorescence to the control treatment (no aphids). Average relative ratios are based on 5 different leaf samples per treatment. (C) Images taken 24 hours after exposure to *M*. *persicae*, *M*. *cerasi and R*. *padi* moults using a laser confocal microscope and processed in ImageJ to generate graph bars representing relative fluorescence to the control treatment (no aphids). Graph indicates fluorescence ratios of leaf samples exposed to moults compared to a no moult control (indicated by C). (D) Levels of ROS in Arabidopsis detached leaves 24 hours after exposure to moults from the aphid species *M*. *persicae*, *M*. *cerasi* and *R*.*padi*. Five moults were placed on each leaf and leaves were collected after 24 hours. Green fluorescence generated by the dye DCFH-DA (dichlorodihydro-fluorescein diacetate) upon contact with ROS was analyzed using confocal microscope. Scale bar 200 μm. Two independent biological replicates were carried out, with 5 samples per treatment per replicate.
(TIF)

S10 Fig Production of ROS in *Arabidopsis* NADPH-oxidase mutants upon aphid interaction. Leaves exposed to different aphid species were incubated with the dye DCFH-DA (dichlorodihydro-fluorescein diacetate) to compare ROS levels during host and nonhost interactions. (A) Levels of reactive oxygen species (ROS) in detached leaves from Arabidopsis Col-0, and the *atrbohD-3* and *atrbohF-3* mutant lines after exposure to aphids species *Myzus persicae*, *M*. *cerasi* and *Rhopalosihpum padi*. Five adult aphids were placed on each leaf and leaves were collected after 3 and 24 hours. Figure shows example images and all 5 images per treatment per timepoint are available in DRYAD (dryad.18b29) (B) and (C) Replicates of the experiment shown in Fig 8. The graphs indicate relative fluorescence of leaf samples exposed to aphids compared to a no aphid control (indicated by C). Average relative ratios are based on 5 different leaf samples per treatment. Mc indicates *Myzus cerasi*, Mp indicates *M*. *persicae* and Rp indicates *Rhopalosiphum padi*. Three independent replicated experiments where performed, with 5 leaf samples per treatment per replicate.
(TIF)

S1 Table Set of 874 *Arabidopsis* genes differentially expressed during host and nonhost interactions with aphids. Among the 874 genes that displayed significant differential expression in at least one of the aphid treatments, three main gene clusters were identified based on their gene expression profiles. The array probe number, normalized expression value (fold changes were log2), TIGR ID number, *Arabidopsis thaliana* accession number, the gene bank accession are given for each gene that showed a differential expression. Putative function and Gene Ontology (GO) annotations, available in the TAIR database, are also detailed for each gene.
(XLSX)

S2 Table Gene Ontology enrichment analyses for the 874 genes differentially expressed across aphid interactions. The Gene Ontology (GO) and the Munich Information Center for Protein Sequences (MIPS) annotations and the terms related to these annotations are mentioned in the first and the second column. The frequency of these GO/MIPS in our set and in the whole-annotated genome, p-values and genes mapped in these groups are also given.
(XLSX)

S3 Table Genes differentially up- and down-regulated per aphid treatment and timepoint. The array probe number, normalized expression value (fold changes were log2), TIGR ID number, *Arabidopsis thaliana* accession number, the gene bank accession are given for each gene that showed a differential expression. Putative function and Gene Ontology (GO) annotations, availabled in the TAIR database, are also detailed for each gene. We marked with a yellow triangle, genes specifically up or down-regulated during the interaction with *M*. *persicae* and showing a similar direction of regulation during the interactions with *M*. *cerasi* and *R*. *padi* by applying a log2 fold change = 0.2 cut off. We marked with a red diamond, genes specifically up or down-regulated during the interaction with *M*. *persicae* and showing a similar direction of regulation during the interactions with *R*. *padi* by applying a log2 fold change = 0.2 cut off.
(XLSX)

S4 Table Genes similarly affected by all aphid treatments per timepoint. The array probe number, normalized expression value (fold changes were log2), TIGR ID number, *Arabidopsis thaliana* accession number, and the gene bank accessions are given for each gene that showed a differential expression.
(XLSX)

S5 Table Gene Ontology enrichment analyses for the genes similarly affected by all aphid treatments per timepoint. The Gene Ontology (GO) and the Munich Information Center for Protein Sequences (MIPS) annotations and the terms related to these annotations are mentioned in the first and the second column. The frequency of these GO/MIPS in our set and in the whole-annotated genome, p-values and genes mapped in these groups are also given.
(XLSX)

S6 Table Gene Ontology enrichment analyses for genes specifically deregulated by *M*.*persicae*, *M*. *cerasi* or *R*. *padi*. The Gene Ontology (GO) and the Munich Information Center for Protein Sequences (MIPS) annotations and the terms related to these annotations are mentioned in the first and the second column. The frequency of these GO/MIPS in our set and in the whole-annotated genome, p-values and genes mapped in these groups are also given.
(XLSX)

S7 Table Set of 96 *Arabidopsis* genes with opposite expression profiles during host, poor-host, or nonhost interactions. 96 selected genes, from the volcano filtering and ANOVA analyses, showing an opposite expression patterns during host, poor-host, or nonhost interactions. +,- and = indicate respectivelly genes up- or down-regulated or unaffected during interaction with *Myzus cerasi* (Mc), *M*. *persicae* (Mp) and *Rhopalosiphum padi* (Rp). The array probe number, normalized expression value (fold changes were log2), TIGR ID number, *Arabidopsis thaliana* accession number, the gene bank accession are given for each gene that showed a differential expression.
(XLSX)

S8 Table Set *Arabidopsis* genes up- or down-regulated during interaction with *M*. *persicae*, *M*. *cerasi* or *R*. *padi*. Classification of genes according to their expression profiles among treatments, performed by volcano plot filtering (fold change ≥ 2.0, t-test p-value ≤ 0.05). The array probe number, normalized expression value (fold changes were log2), TIGR ID number, Arabidopsis thaliana accession number, the gene bank accession are given for each gene that showed a differential expression. Putative function and Gene Ontology (GO) annotations, availabled in the TAIR database, are also detailed for each gene.
(XLSX)

S9 Table Set *Arabidopsis* genes up- or down-regulated or unaffected during interaction with *M*. *persicae*, *M*. *cerasi* or *R*. *padi*. Classification of the 874 genes according to their expression profiles among treatments (fold change ≥ 2.0, t-test p-value ≤ 0.05). The array probe number, normalized expression value (fold changes were log2), TIGR ID number, Arabidopsis thaliana accession number, the gene bank accession are given for each gene that showed a differential expression. Putative function and Gene Ontology (GO) annotations, available in the TAIR database, are also detailed for each gene.
(XLSX)

S10 Table Gene Ontology enrichment analyses for the 96 genes with opposite expression profiles during host, poor-host, or nonhost interactions. The Gene Ontology (GO) and the Munich Information Center for Protein Sequences (MIPS) annotations and the terms related to these annotations are mentioned in the first and the second column. The frequency of these GO/MIPS in our set and in the whole-annotated genome, p-values and genes mapped in these groups are also given.
(XLSX)

S11 Table Primers used for the RT-qPCR validation. Name, description and sequence are given for each primer used.
(XLSX)

S12 Table Knock-out lines used for the infestation tests. NASC ID number, name of the lines and description are given for each knock-out line.
(XLSX)
