# Supplementary figures and images for: Characterization of Arabidopsis Transcriptional Responses to Different Aphid Species Reveals Genes that Contribute to Host Susceptibility and Non-host Resistance
Source: PLoS Pathog. 2015 May 20;11(5):e1004918. doi: 10.1371/journal.ppat.1004918 (PMC4439036; doi:10.1371/journal.ppat.1004918)

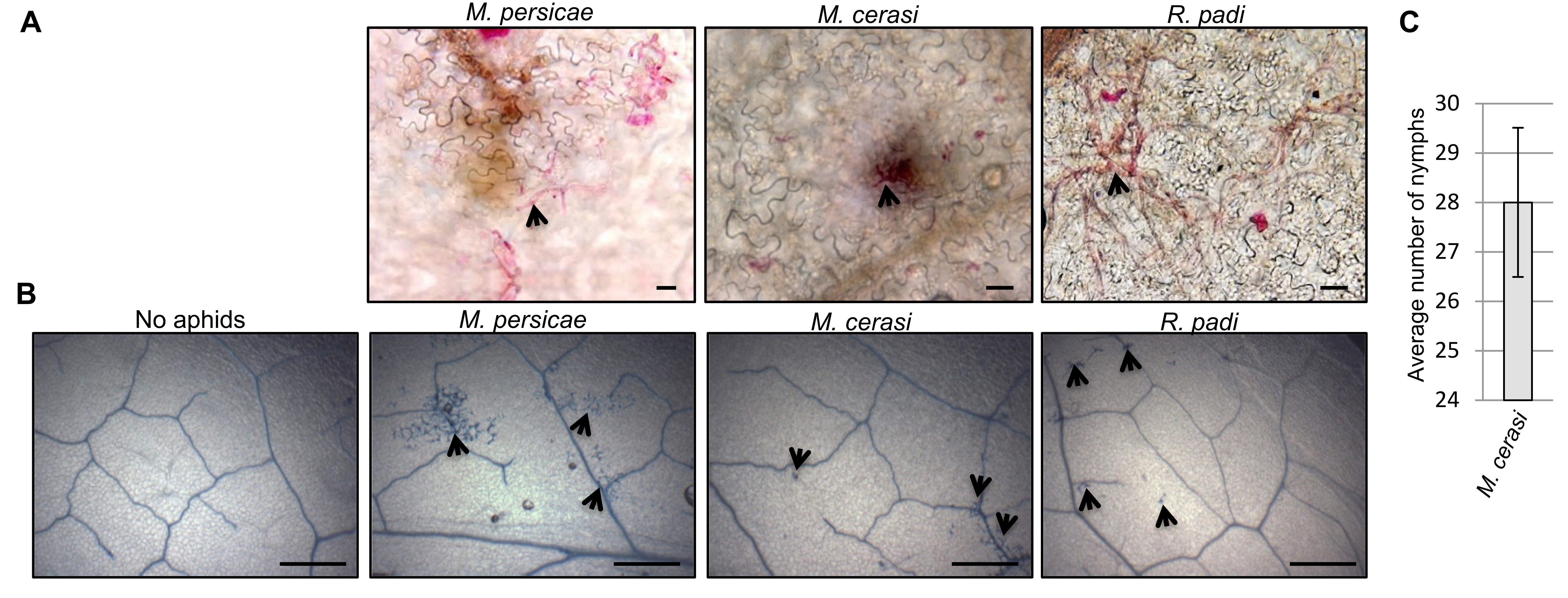

Supplement: S1 Fig — Fuchsin stain (A) and Trypan blue stain (B) of Arabidopsis leaves exposed to different aphids species. Scale bars 20 μm (A). Scale bars 20mm (B). Fuchsin staining visualized aphid stylet pathways, whereas Trypan blue staining visualized cell death, as indicated by the arrows. (C) Aphid colonization of American cress by Myzus cerasi. Graph shows the mean number of nymphs produced after two weeks on cress plants. Error bars indicate standard error. Three independent biological replicates were carried out, with 4 plants per replicate. (TIF) [file ppat.1004918.s001.tif]

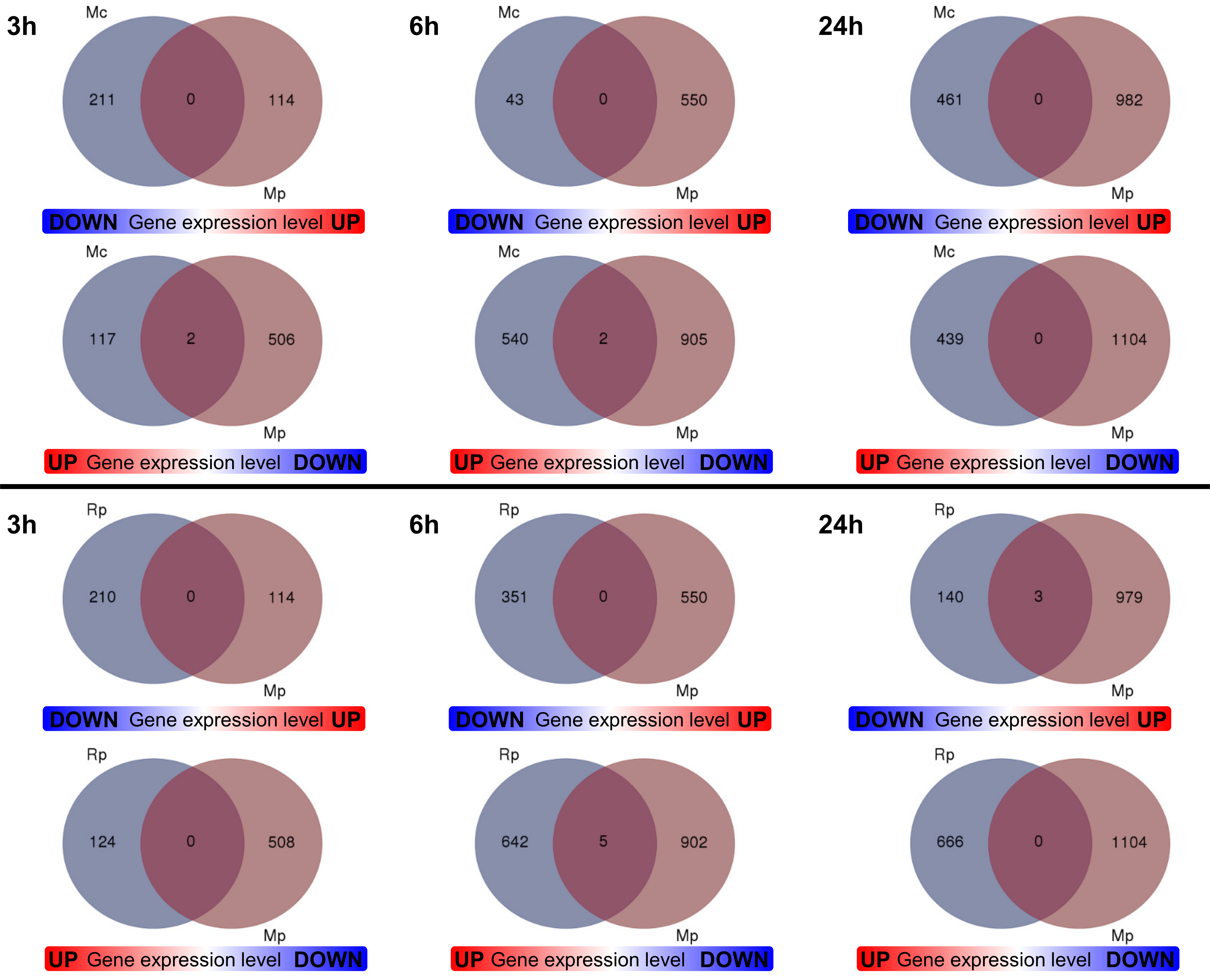

Supplement: S2 Fig — Volcano filtering was used to identify gene with opposite gene expression profiles among treatments. Numbers are numbers of genes identified using statistical analyses of aphid treatment per timepoint versus the non-aphid control (p-value<0.05). Mc indicates Myzus cerasi, Mp indicates M. persicae and Rp indicates Rhopalosiphum padi. (TIF) [file ppat.1004918.s002.tif]

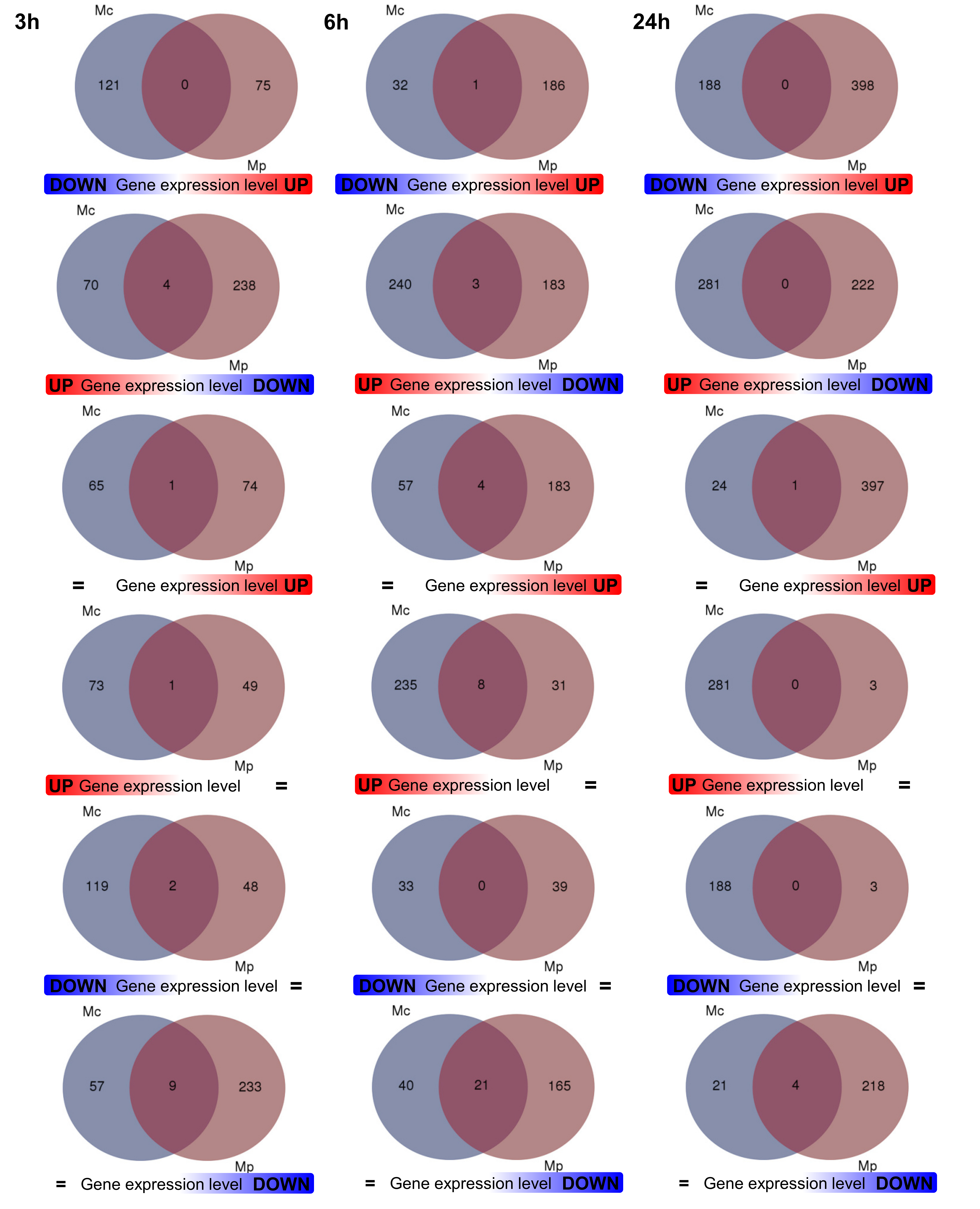

Supplement: S3 Fig — Of the 874 genes in total identified as differentially expressed using one-way ANOVA with Bonferroni correction (p-value<0.05), we generated Venn diagrams to perform pairwise comparisons and identify genes differentially expressed across interactions. Mp indicates Myzus persicae and Mc indicates Myzus cerasi. Data corresponds to S9 Table. (TIF) [file ppat.1004918.s003.tif]

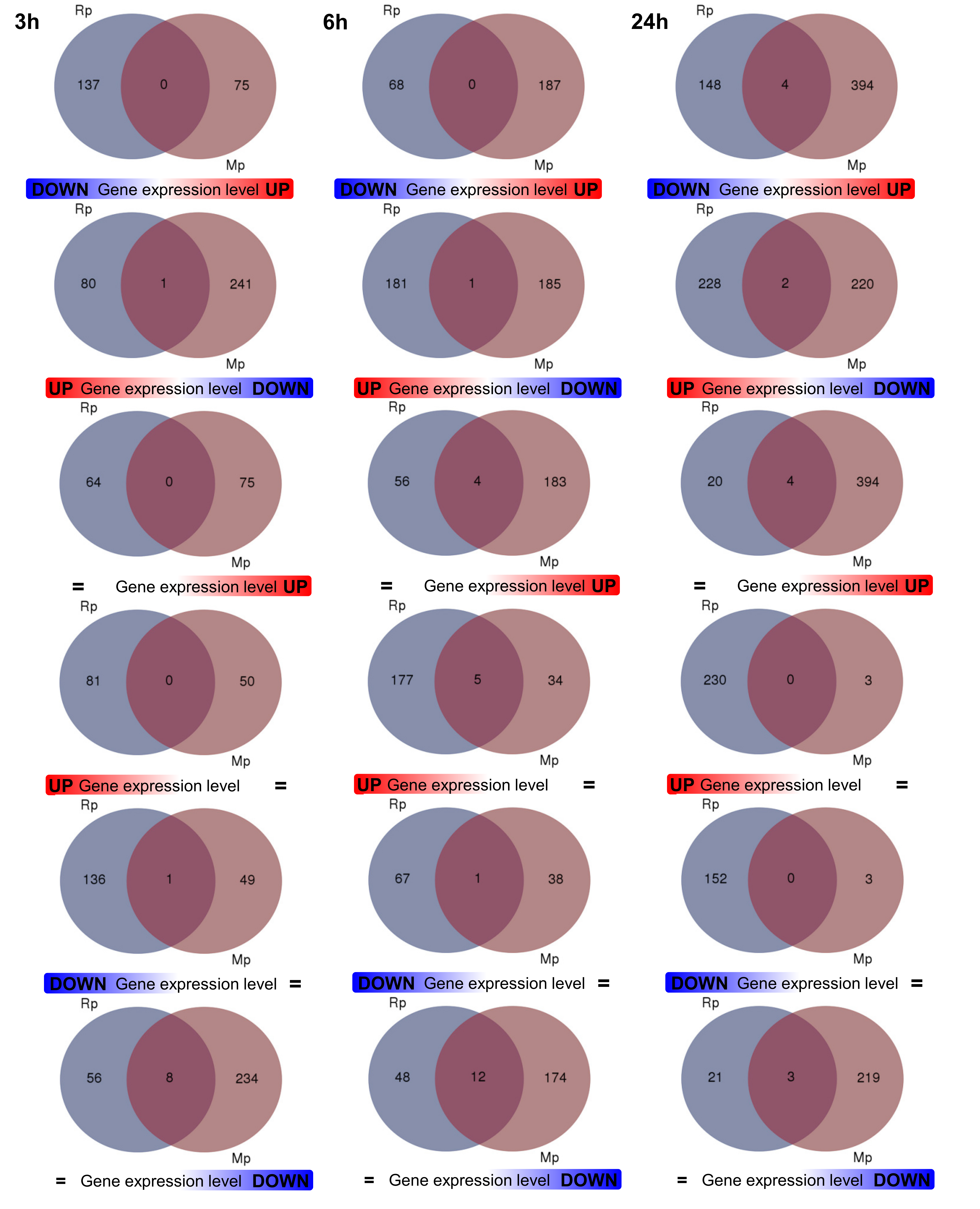

Supplement: S4 Fig — Of the 874 genes in total identified as differentially expressed using one-way ANOVA with Bonferroni correction (p-value<0.05), we generated Venn diagrams to perform pairwise comparisons and identify genes differentially expressed across interactions. Mp indicates Myzus persicae and Rp indicates Rhopalosiphum padi. Data corresponds to S9 Table. (TIF) [file ppat.1004918.s004.tif]

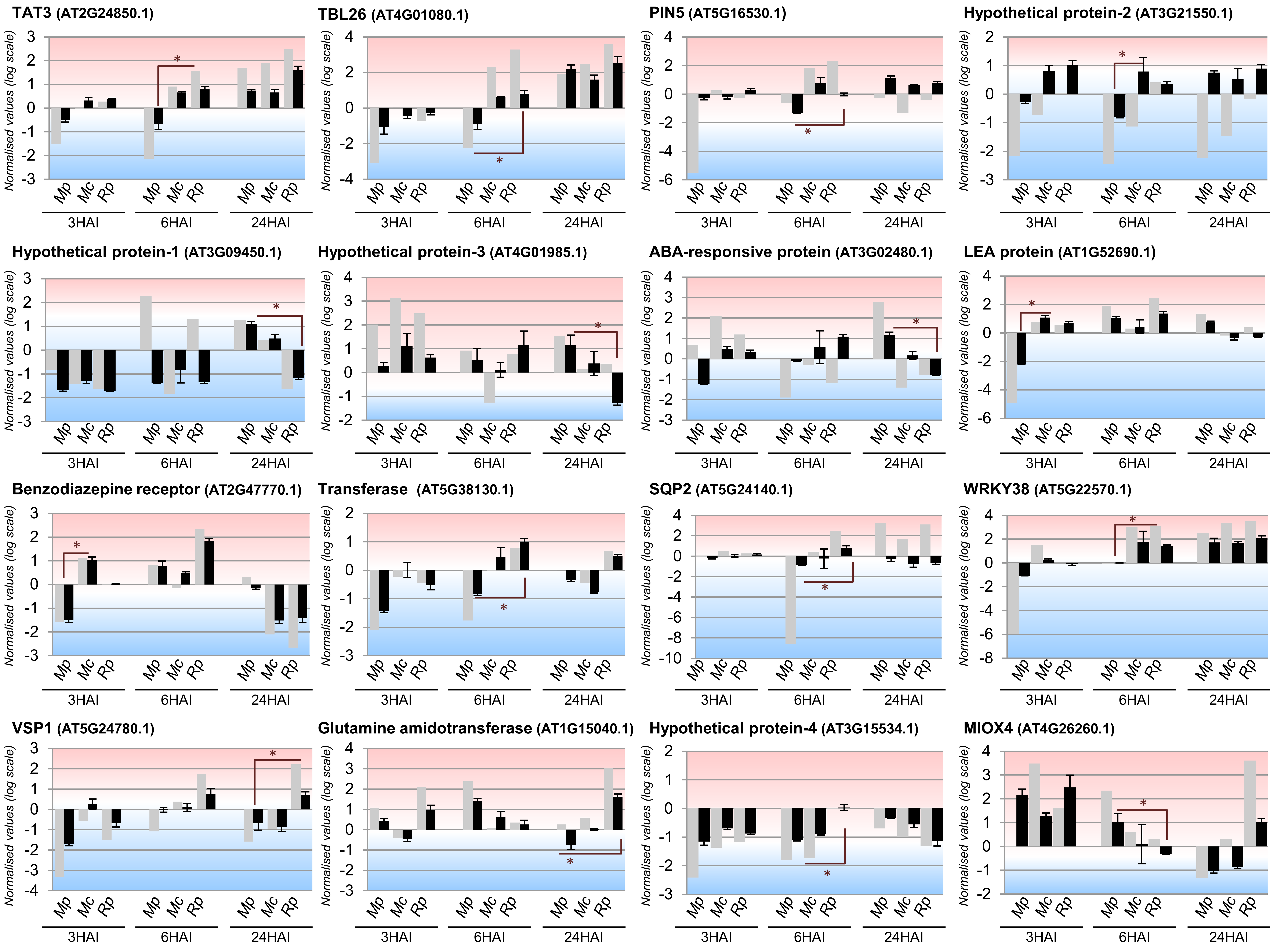

Supplement: S5 Fig — Gene expression profiles of selected candidate genes were evaluated using RT-qPCR. Intensity values represent the average of the Log2 (ratio = Esample ΔCtsample/ Ecalibrator ΔCtcalibrator as calibrator, housekeeping genes ACT2, EF1α and UBQ22). Intensity values ± standard deviation were plotted for each gene. Grey bars represent the expression profiles according to the RT-qPCR results. Black bars represent the average expression profiles according to the microarrays results, based on three biological replicates, and error bars indicate the standard error. Red lines and stars indicate the comparisons found to be differential among interactions by statistical tests (volcano plot analyses/or ANOVA) based on the microarray data. The RT-qPCR experiments were performed on the pooled samples of the three biological replicates. (TIF) [file ppat.1004918.s005.tif]

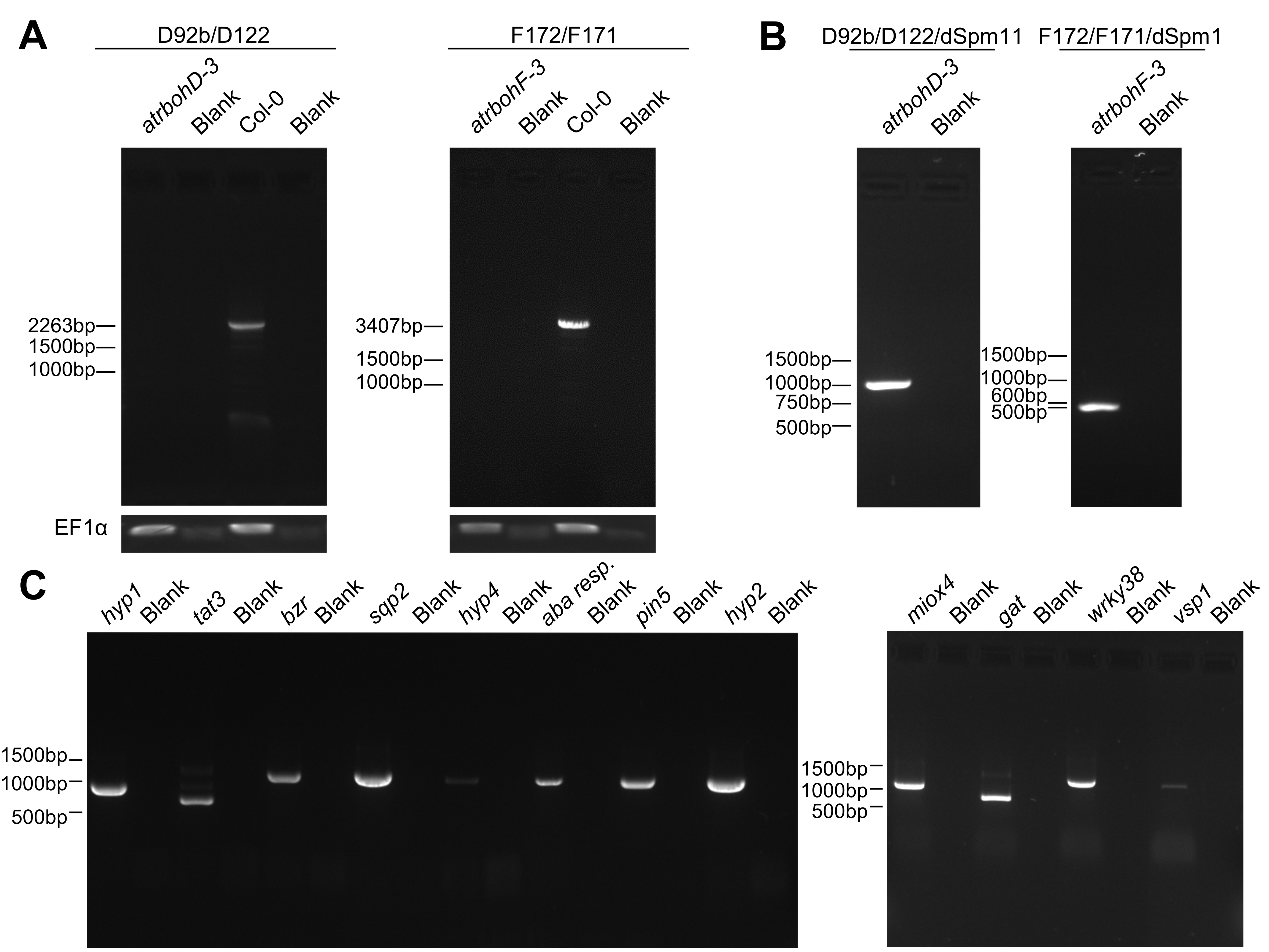

Supplement: S6 Fig — (A) The expression of AtRbohD and AtRbohF was evaluated by RT-PCR in cDNA from the atrbohD-3 and atrbohF-3 knockout mutants and wild-type Col-0 plants. (B) Transposon insertions were confirmed by PCR on atrbohD-3 and atrbohF-3 genomic DNA. (C) T-DNA insertion confirmation by PCR on genomic DNA of all knock-out lines used in this article. (TIF) [file ppat.1004918.s006.tif]

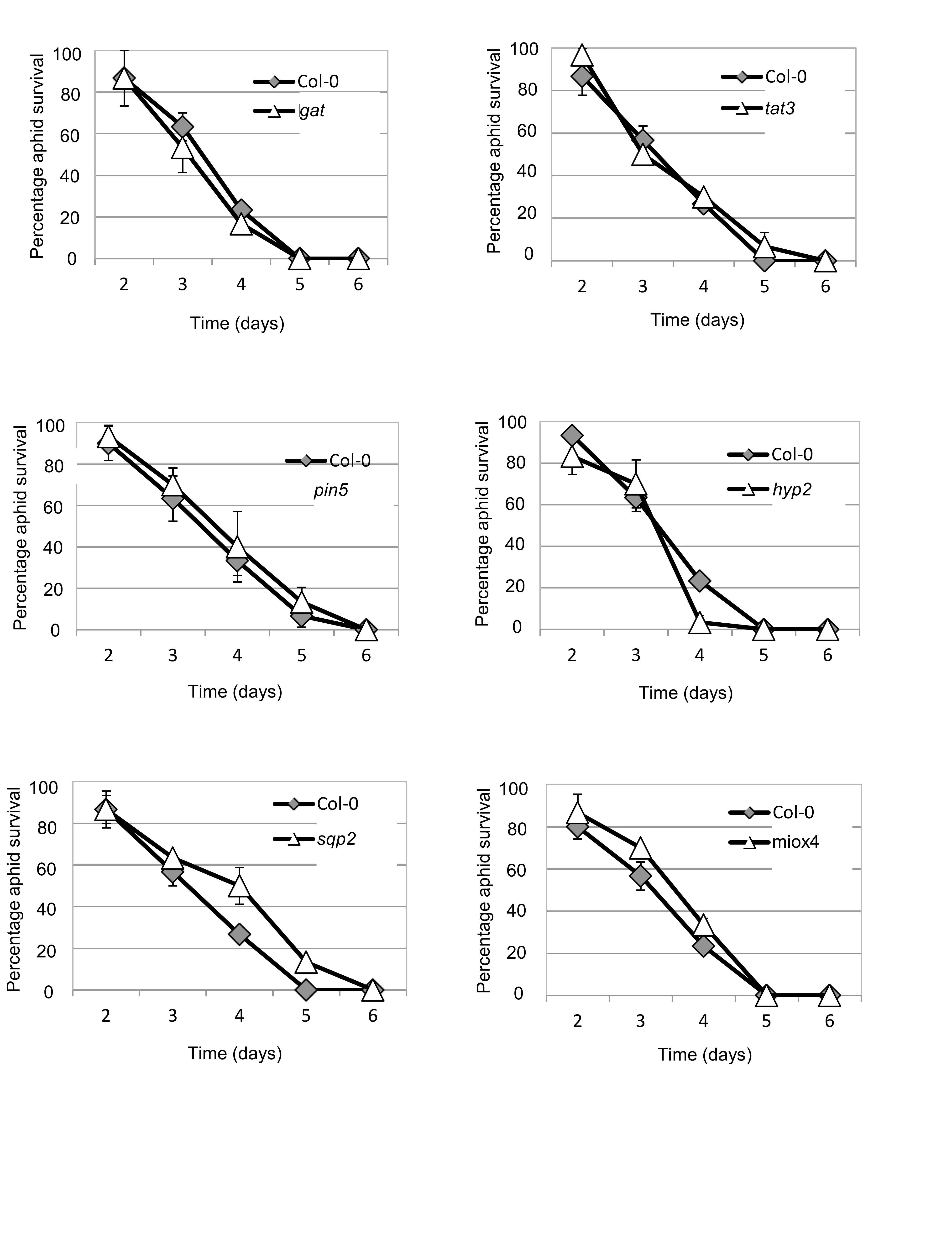

Supplement: S7 Fig — Graphs showing R. padi aphid survival on the knock-out mutants, and the control (Col-0) over 6 days. Five adult aphids were placed on four-week old plants and survival was monitored the following 6 days. Three independent biological replicates were carried out, with 10 plants per replicate. Error bars indicate standard error (TIF) [file ppat.1004918.s007.tif]

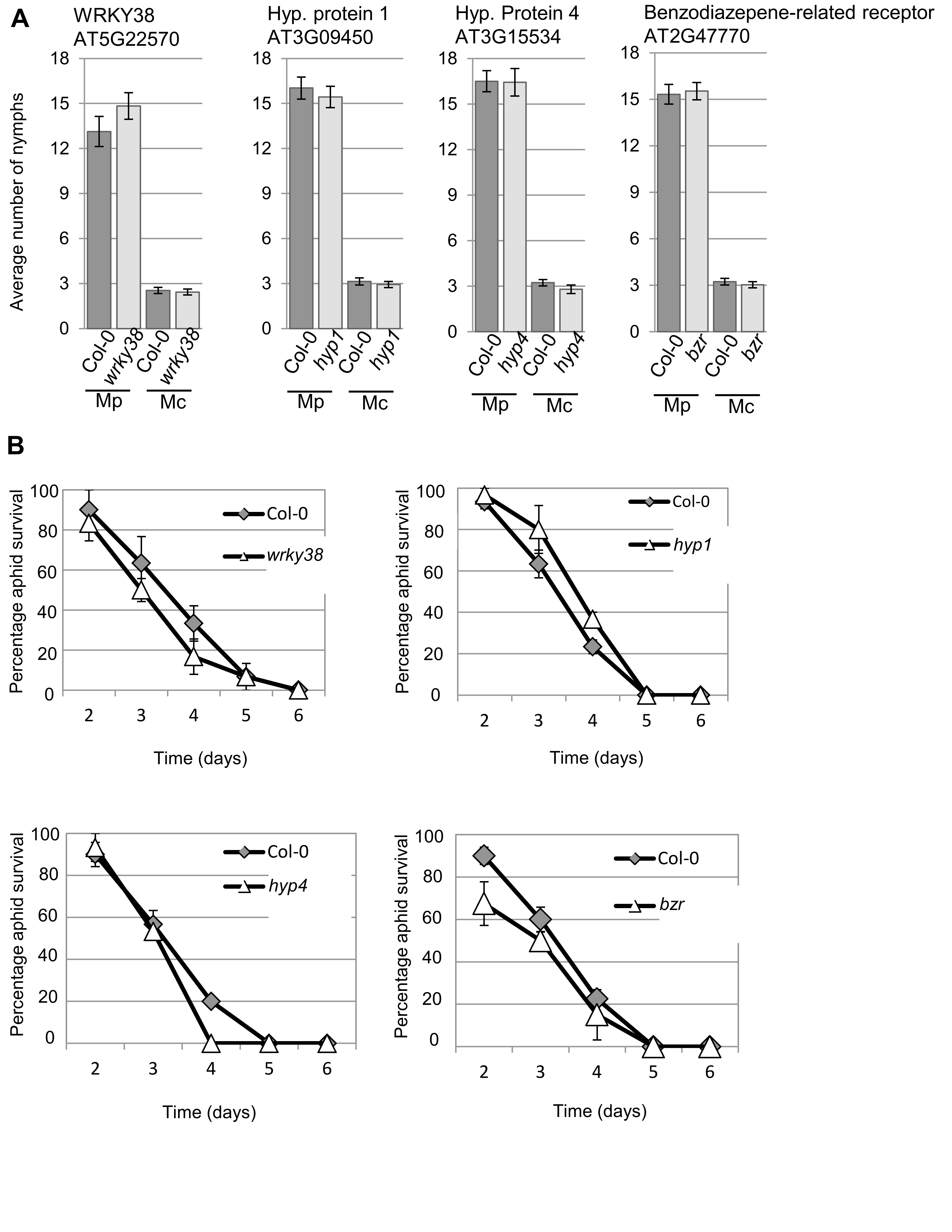

Supplement: S8 Fig — (A) M. persicae and M. cerasi performance on Arabidopsis knock-out mutants and Col-0 wild-type plants. Four-week old plants were exposed to two adult aphids and nymph production was counted after 10 days. Average nymph production was calculated from three independent replicated experiments, with 10 plants per replicate per treatment. (B) Graph showing R. padi aphid survival on the knock mutants and the control (Col-0) over 6 days. Five adult aphids were placed on four-week old plants and survival was monitored the following 6 days. Three independent biological replicates were carried out, with 10 plants per replicate. Error bars indicate standard error. (TIF) [file ppat.1004918.s008.tif]

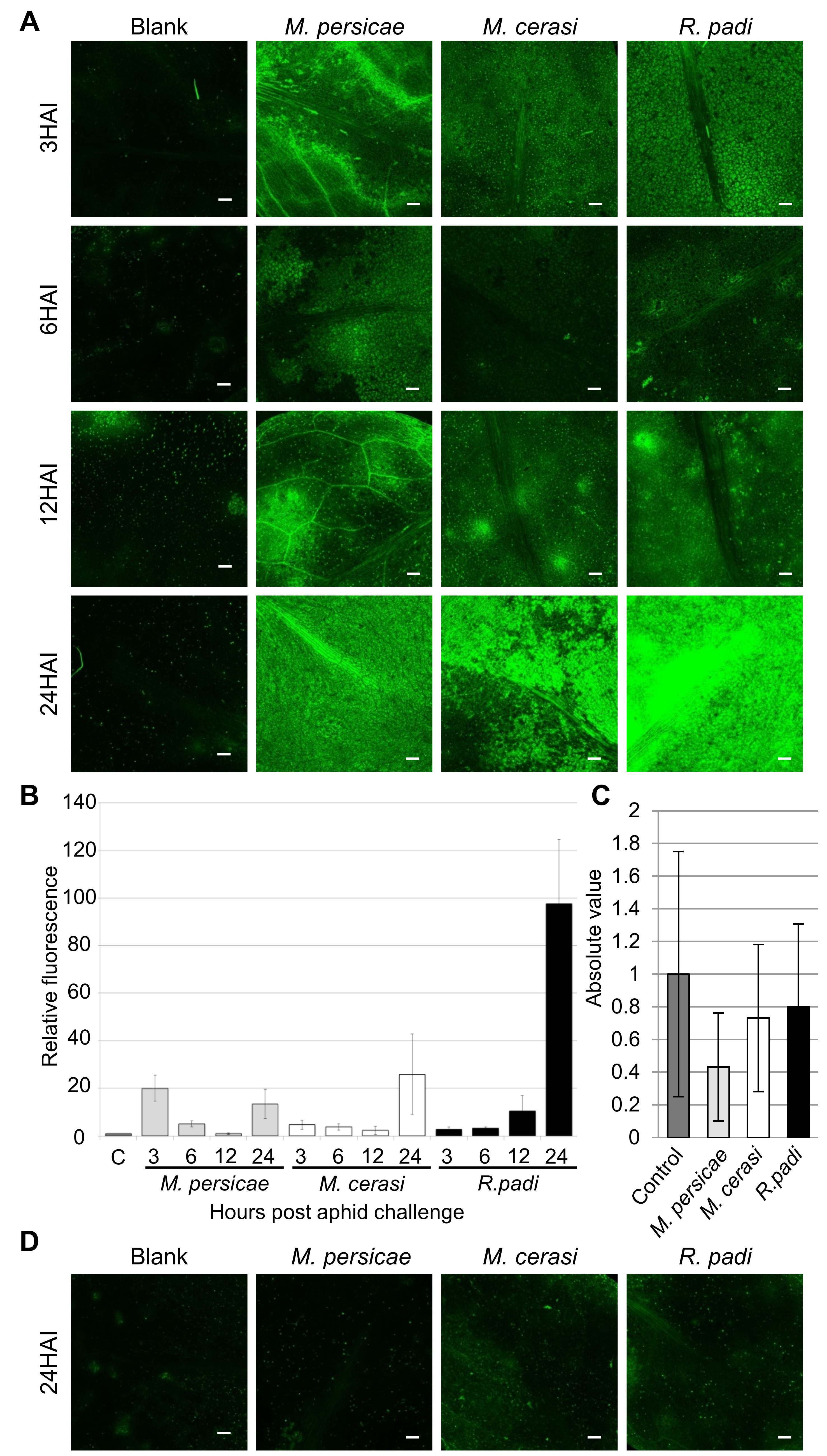

Supplement: S9 Fig — Leaves exposed to different aphid species were incubated with the dye DCFH-DA (dichlorodihydro-fluorescein diacetate) to compare ROS levels during host and non-host interactions. (A) Levels of ROS in Arabidopsis detached leaves after Myzus persicae, M. cerasi and Rhopalosiphum padi exposure. Five adult aphids were placed on each leaf and leaves were collected after 3, 6, 12 and 24 hours. Images were taken with a laser confocal microscope. Figure shows example images and all 5 images per treatment per timepoint are available in DRYAD (dryad.18b29) (B) Second replicate of the experiment shown in Fig 7. Images were taken using a laser confocal microscope and processed in ImageJ to generate graph bars representing relative fluorescence to the control treatment (no aphids). Average relative ratios are based on 5 different leaf samples per treatment. (C) Images taken 24 hours after exposure to M. persicae, M. cerasi and R. padi moults using a laser confocal microscope and processed in ImageJ to generate graph bars representing relative fluorescence to the control treatment (no aphids). Graph indicates fluorescence ratios of leaf samples exposed to moults compared to a no moult control (indicated by C). (D) Levels of ROS in Arabidopsis detached leaves 24 hours after exposure to moults from the aphid species M. persicae, M. cerasi and R.padi. Five moults were placed on each leaf and leaves were collected after 24 hours. Green fluorescence generated by the dye DCFH-DA (dichlorodihydro-fluorescein diacetate) upon contact with ROS was analyzed using confocal microscope. Scale bar 200 μm. Two independent biological replicates were carried out, with 5 samples per treatment per replicate. (TIF) [file ppat.1004918.s009.tif]

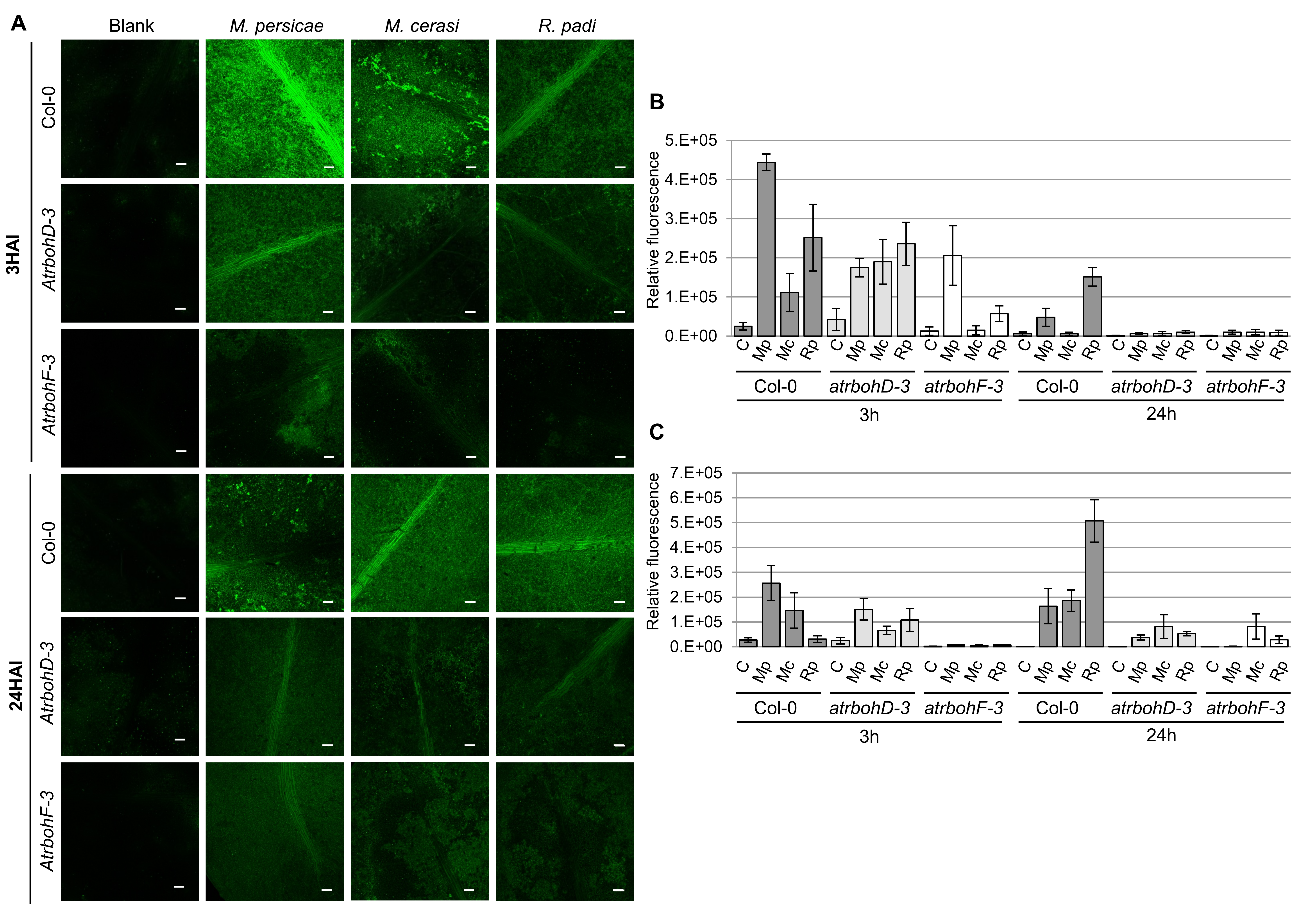

Supplement: S10 Fig — Leaves exposed to different aphid species were incubated with the dye DCFH-DA (dichlorodihydro-fluorescein diacetate) to compare ROS levels during host and nonhost interactions. (A) Levels of reactive oxygen species (ROS) in detached leaves from Arabidopsis Col-0, and the atrbohD-3 and atrbohF-3 mutant lines after exposure to aphids species Myzus persicae, M. cerasi and Rhopalosihpum padi. Five adult aphids were placed on each leaf and leaves were collected after 3 and 24 hours. Figure shows example images and all 5 images per treatment per timepoint are available in DRYAD (dryad.18b29) (B) and (C) Replicates of the experiment shown in Fig 8. The graphs indicate relative fluorescence of leaf samples exposed to aphids compared to a no aphid control (indicated by C). Average relative ratios are based on 5 different leaf samples per treatment. Mc indicates Myzus cerasi, Mp indicates M. persicae and Rp indicates Rhopalosiphum padi. Three independent replicated experiments where performed, with 5 leaf samples per treatment per replicate. (TIF) [file ppat.1004918.s010.tif]
